# Characterization of volatile organic compounds with anti-atherosclerosis effects in *Allium macrostemon* Bge. and *Allium chinense* G. Don by head space solid phase microextraction coupled with gas chromatography tandem mass spectrometry

**DOI:** 10.3389/fnut.2023.996675

**Published:** 2023-02-01

**Authors:** Zifei Qin, Shuyi Duan, Yuan Li, Xinqiang Li, Han Xing, Zhihong Yao, Xiaojian Zhang, Xinsheng Yao, Jing Yang

**Affiliations:** ^1^Department of Pharmacy, The First Affiliated Hospital of Zhengzhou University, Zhengzhou, China; ^2^Henan Applied and Translational Center of Precision Clinical Pharmacy, Zhengzhou, China; ^3^College of Pharmacy, Jinan University, Guangzhou, China; ^4^Department of Pathology, The First Affiliated Hospital of Zhengzhou University, Zhengzhou, China

**Keywords:** *Allium macrostemon* Bge., *Allium chinense* G. Don, volatile organic compounds, anti-atherosclerosis effects, differentially expressed components

## Abstract

**Introduction:**

*Allium macrostemon* Bge. (AMB) and *Allium chinense* G. Don (ACGD) are both edible *Allium* vegetables and named officinal Xiebai (or Allii Macrostemonis Bulbus) in East Asia. Their medicinal qualities involve in lipid lowering and anti-atherosclerosis effects. And steroidal saponins, nitrogenous compounds and sulfur compounds are like the beneficial components responsible for medicinal functions. Sulfur compounds are the recognized main components both in the volatile oils of AMB and ACGD. Besides, few researches were reported about their holistic chemical profiles of volatile organic compounds (VOCs) and pharmacodynamic effects.

**Methods:**

In this study, we first investigated the lipid-lowering and anti-atherosclerotic effects of volatile oils derived from AMB and ACGD in *ApoE*^–/–^ mice with high fat and high cholesterol diets.

**Results:**

The results showed the volatile oils of AMB and ACGD both could markedly reduce serum levels of TG, TC, and LDL-C (*p* < 0.05), and had no alterations of HDL-C, ALT, and AST levels (*p* > 0.05). Pathological results displayed they both could obviously improve the morphology of cardiomyocytes and the degree of myocardial fibrosis in model mice. Meanwhile, oil red O staining results also proved they could apparently decrease the lesion areas of plaques in the aortic intima (*p* < 0.05). Furthermore, head space solid phase microextraction coupled with gas chromatography tandem mass spectrometry combined with metabolomics analysis was performed to characterize the VOCs profiles of AMB and ACGD, and screen their differential VOCs. A total of 121 and 115 VOCs were identified or tentatively characterized in the volatile oils of AMB and ACGD, respectively. Relative-quantification results also confirmed sulfur compounds, aldehydes, and heterocyclic compounds accounted for about 85.6% in AMB bulbs, while approximately 86.6% in ACGD bulbs were attributed to sulfur compounds, ketones, and heterocyclic compounds. Multivariate statistical analysis showed 62 differentially expressed VOCs were observed between AMB and ACGD, of which 17 sulfur compounds were found to be closely associated with the garlic flavor and efficacy.

**Discussion:**

Taken together, this study was the first analysis of holistic chemical profiles and anti-atherosclerosis effects of AMB and ACGD volatile oils, and would benefit the understanding of effective components in AMB and ACGD.

## Graphical Abstract

Analytical flow chart of chemical and pharmacological similarities and differences of AMB and ACGD. 
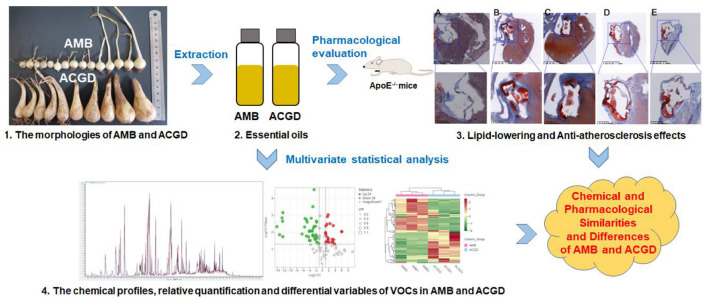

## Introduction

Allii Macrostemonis Bulbus, also called officinal Xiebai in East Asia, exhibits various beneficial health properties including lipid lowering, anti-atherosclerosis, myocardial protection, anti-depressant, anti-bacterial effects (1–4). Xiebai is an edible and medicinal herb, which was first recorded in Divine Farmer’s Classic of Materia Medica (aka Shennong Bencao Jing), and it has two sources of medicinal herbs, *Allium macrostemon* Bge. (AMB) and *Allium chinense* G. Don (ACGD) (1). In clinical application, the dried bulbs of AMB or ACGD are traditionally used as the monarch drug and antithrombosis agents to treat coronary heart disease in combination with other medicinal herbs (*Trichosanthis fructus*, *Pinelliae rhizome*, etc.) for over two thousands of years in East Asia (5). In addition, Xiebai is also listed in the catalog that are suggested to be used as food and medicine by National Health Commission (6). AMB and ACGD both as edible *Allium* vegetables, are often cooked with other ingredients or pickled as a pleasing seasoning. And they are widely distributed in East Asia, and also naturalized in other parts of Asia as well as Europe and North America (7, 8). The remarkable medicinal values and health functions attracted increasing attentions on the effective components of AMB and ACGD.

Steroidal saponins, sulfur compounds, nitrogenous compounds, flavonoids, polysaccharides in AMB and ACGD are like the main contributors to the pharmacological effects (1, 9–11). Recently, network pharmacology analysis showed these components act on multiple signal pathways through synergistic effects (12). So far, the chemical similarities and differences of the dried bulbs of AMB and ACGD have been successfully deciphered including chemical profiles, content levels and metabolic pathways (1, 10, 13–15). For the fresh bulbs of AMB and ACGD, sulfur-containing compounds are their main volatile organic compounds (VOCs), especially dimethyl trisulfide, methyl propyl disulfide, methyl allyl trisulfide, dimethyl disulfide (16–18). These sulfur VOCs are same or similar with those in garlic oils (*Allium sativum* L.), a famous lipid lowering agent (19). And among over 600 *Allium* species, AMB, ACGD, and garlic are all as food and herbs, and this is also a rare phenomenon for alliaceous species (1, 19). However, except for individual sulfur compounds, fewer researches were reported on the holistic chemical profiles of VOCs in AMB and ACGD fresh bulbs.

Another interesting phenomenon is that the fresh bulbs of AMB are commonly processed and used as the medicinal herbs of Xiebai in China, while the fresh bulbs of ACGD are usually processed into food products in Asia countries and North America (7, 19, 20). At present, about 5,000 tons of fresh AMB bulbs are consumed per year for medicinal purposes, whereas approximately 300,000 tons of fresh ACGD bulbs per year are pickled and canned as vegetables, and exported to Japan, Korea, Southeast Asia, and North America (21, 22). So far, several researches had reported that the volatile oils of ACGD showed protecting histopathological liver alteration and insecticidal activity (16, 23), while the volatile oils of AMB exhibited vasodilation effect and larvicidal activity (17, 24). However, other pharmacological effects related to their traditional efficacy of AMB and ACGD volatile oils including lipid lowering and anti-atherosclerosis were rarely reported so far. These also aroused our concerns about their similarities and differences on prevention and treatment of atherosclerosis, as well as their chemical and content consistency of holistic VOCs in AMB and ACGD.

For these goals, we first investigated the lipid lowering and anti-atherosclerosis activities of volatile oils derived from AMB and ACGD in *ApoE*^–/–^ mice with high fat and high cholesterol diets. Further, the holistic VOCs were identified and tentatively characterized in AMB and ACGD bulbs based on retention times, retention index (RI) and match factor (MF) by GC-MS combined with NIST20 and MWGC database. In addition, multivariate statistical analysis including principal component analysis (PCA), hierarchical cluster analysis (HCA), and orthogonal partial least squares discriminant analysis (OPLS-DA) were performed to identify the significantly regulated VOCs between AMB and ACGD oils. Moreover, relative-quantification of these VOCs were carried out based on internal standard methods, followed remarkable visual content differences of several differential VOCs in these two herbs. Taken together, this study would increase our understanding about the holistic VOCs profile and anti-atherosclerosis effects of AMB and ACGD.

## Materials and methods

### Chemicals and materials

Alanine aminotransferase (ALT) assay kit, aspartate aminotransferase (AST) assay kit, total cholesterol (TC) assay kit, triglyceride (TG) assay kit, low-density lipoprotein cholesterol (LDL-C) assay kit, high-density lipoprotein cholesterol (HDL-C) assay kit were all purchased from Nanjing Jiancheng Bioengineering Institute (Nanjing, China). Rosuvastatin calcium tablets (RSV) were obtained from Chia-Tai Tianqing Pharmaceutical Group Co., Ltd (Nanjing, China). Purified atherosclerotic model diets with 1.25% cholesterol (XT108C) and control diets without cholesterol (XT102C) were purchased from Jiangsu Synergetic Pharmaceutical Bioengineering Co., Ltd (Nanjing, China). Other chemicals and reagents were of analytical grade or better.

**FIGURE 1:**
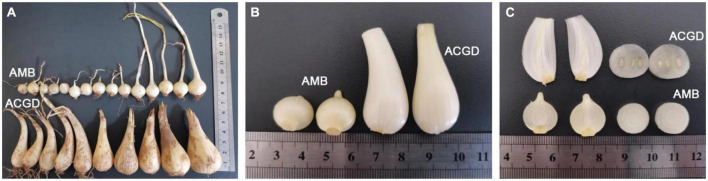
The overall morphology **(A)**, locally enlarged phenotype **(B)**, and cross section and longitudinal section **(C)** of fresh bulbs of *Allium macrostemon* Bge. (AMB) and *Allium chinense* G. Don (ACGD).

The fresh bulbs of *Allium macrostemon* Bge. (AMB) and *Allium chinense* G. Don (ACGD) were collected from Shenyang in Liaoning province and Xinjian in Jiangxi province of China, respectively. The morphologies of these two vegetables are obviously different (Figure 1A). The fresh bulbs of AMB are irregularly circular or ovoid (0.5–2.0 cm high and 0.3–1.5 cm in diameter) and externally yellowish-white or pale, yellowish-brown while the fresh bulbs of ACGD are lightly compressed long-ovate (2.0–5.0 cm high and 0.5–3.0 cm in diameter) and externally pale yellowish-brown or brown (Figure 1B). The developing flower buds in ACGD are significantly more than those in AMB (Figure 1C). The bulbs were taxonomically identified by Professor Xinsheng Yao who works in the College of Pharmacy, Jinan University, China. And their surface was disinfected with 75% alcohol, and immediately frozen in liquid nitrogen, and stored at −80^°^C before use.

### Animal experiments

Male C57BL/6 mice (*n* = 8) and *ApoE*^–/–^ mice (*n* = 32) (both 8–10 weeks, 20 ± 2 g weight) were obtained from Zhengzhou Chuangsheng Biological Engineering Co., Ltd (Zhengzhou, China), and fed in the Experimental Animals Center of Zhengzhou University. The animal protocols were approved and conducted based on the guidelines of Laboratory Animal Ethics Committee of Zhengzhou University. All procedures were in accordance with the Guide for the Care and Use of Laboratory Animals (National Institute of Health). The mice were kept in an animal room at (23 ± 2)^°^C and (60 ± 5)% relative humidity under a 12 h dark/light cycle. They had free access to water and food for a week before experiments.

The wild-type C57BL/6 mice (*n* = 8) was set as control group and fed with control diets. And *Apo*^–/–^ mice were all fed with model diets and divided into four groups as follows, model group (*n* = 8), RSV group (rosuvastatin, *n* = 8), AMB group (volatile oils of AMB, *n* = 8) and ACGD group (volatile oils of ACGD, *n* = 8). The intragastric dosage for RSV group was 2.0 mg/kg/day, while the dosages for AMB group and ACGD group were 100.0 mg/kg/day (25–27). During high fat and high cholesterol modeling for 12 weeks, *ApoE*^–/–^ mice were simultaneously treated with RSV, volatile oils of AMB and volatile oils of ACGD at the same time, respectively. Before experiments, all mice were fasted overnight. Blood samples were received through the extraction of eyeball method, and serum samples were prepared for the analysis of biochemical indicators. In addition, aortic tissues were obtained and fixed in 4% paraformaldehyde in phosphate buffered saline (PBS). And they were further embedded in paraffin for histopathological analysis including hematoxylin-eosin (HE) staining, Masson staining and Oil Red O staining.

### Sample preparation

The volatile oil of AMB and ACGD were prepared and obtained by steam distillation. The fresh bulbs of AMB or ACGD (200 g) were crushed and placed in round bottom flask. And the samples were soaked by 1,000 mL water for overnight. The mixture was heated and refluxed for 8 h. Collected petroleum ether fractions were distillated, and volatile oils were obtained and weighted. The extraction rates of AMB and ACGD were 0.93 and 0.81%, respectively. These obtained volatile oils were used for animal experiments.

*Allium macrostemon* Bge. and ACGD samples were ground to a powder in liquid nitrogen. The powder (1.0 g) was immediately transferred to a 20 mL head-space vial (Agilent, Palo Alto, CA, USA). NaCl saturated solution (2 mL) and 10 μL of internal solution ([2H8]-acetophenone, 50 μg/mL) were added in head-space vial. NaCl solution was used to inhibit any enzyme reaction. The vials were sealed by crimp-top caps with TFE-silicone headspace septa. At the time of SPME analysis, each vial was placed in 100^°^C for 5 min. And 120 μm divinylbenzene (DVB)/carboxen (CAR)/polydimethylsiloxane (PDMS) extraction fiber was exposed to the headspace of the sample for 15 min at 100^°^C. After sampling, desorption of volatiles from the fiber coating was performed in the injection port of 7890B-7000D apparatus (Agilent, Palo Alto, CA, USA) at 250^°^C for 5 min in the splitless mode.

### GC-MS conditions

The separation of volatile organic compounds (VOCs) was carried out on a DB-5MS (5% phenyl polymethylsiloxane) capillary column (30 m × 0.25 mm × 1.0 μm) by a 7890B GC and 7000D mass spectrometer (Agilent, CA, USA). Helium (purity not less than 99.999%) was used as the carrier gas at a linear velocity of 1.2 mL/min. The injector temperature was at 250^°^C and the samples were injected in the spitless mode with 3.5 min delay of the solvents. The gradient elution program for temperature was as follows: keeping 40^°^C for 3.5 min, increasing at 10^°^C/min to 100^°^C, at 7^°^C/min to 180^°^C, at 25^°^C/min to 280^°^C, holding 280^°^C for 5 min.

Mass spectra was collected in electron impact (EI) ionization mode. The detailed parameters were as follows. The ion source, quadrupole mass detector and transfer line temperatures were at 230, 150, and 280^°^C, respectively. The electron energy was set at 70^°^eV. The full scanning mass ranges were at *m/z* 50–400 amu at 1 s intervals. All data were collected and processed using MassHunter software (Agilent, CA, USA).

### Qualitative and quantitative analysis

The obtained offline original data were browsed and processed by qualitative and quantitative analysis workflows under the manipulation system of MassHunter (Agilent, CA, USA). Identification and quantification of VOCs were achieved based on retention time behaviors, mass spectra and linear retention index of volatile compounds in the NIST20 database and MWGC data library (Wuhan Metware Biotechnology Co. Ltd., Wuhan, China). The match factors were calculated and obtained after the mass spectrum fingerprint of VOCs was matched with the reference fingerprint by mirror comparison (28, 29). The match factor over 70.0 suggested more accurate identification of VOCs in volatile oils, while the characterization of VOCs was for reference when the match factor was less than 70.0.

For quantitative analysis of VOCs, the mass concentrations could be calculated with reference to the internal standard method using Equation (1) as follow (30). C_voc_ and C_is_ are the mass concentrations of identified VOCs and the internal standard ([2H8]-acetophenone, 50 μg/mL), respectively. Similarly, A_voc_ and A_is_ are the chromatographic peak areas of identified VOCs and internal standard, respectively.


(1) $${\text{C}}_{\text{voc}}=\left({\text{C}}_{\text{is}}\times {\text{A}}_{\text{voc}}\right)/{\text{A}}_{\text{is}}$$

### Multivariate statistical analysis

Unsupervised principal component analysis (PCA) was performed after the original data was unit variance scaled (31). The hierarchical cluster analysis (HCA) results of samples and VOCs were presented as heatmaps with dendrograms, and normalized signal intensities of VOCs (unit variance scaling) are visualized as a color spectrum. Variable influence on projection (VIP) were obtained from orthogonal partial least squares discriminant analysis (OPLS-DA) results, which also contained score plots and permutation plots (31). Before OPLS-DA, the obtained data were log transformed (log_2_) and mean centered. And a further permutation test (200 permutations) was carried out to avoid overfitting. The significantly regulated VOCs between AMB and ACGD groups were selected based on the principles of VIP ≥ 1, *p*-value < 0.05, and absolute Log_2_FC (fold change) ≥ 1. Multivariate statistical analysis was performed using the Metware Cloud, a free online platform for data analysis^1^.

### Statistical analysis

Three biological replicates (*n* = 3) were used for GC-MS analysis. And the error bars represented the standards deviations of six determinations (*n* = 8) for animal experiments. The differences between two groups were statistically analyzed using Student’s *t*-test (*p* < 0.05) with GraphPad Prism 5 software (SanDiego, CA). The levels of difference were set at *p* < 0.05 (*), *p* < 0.01 (^**^), and *p* < 0.001 (^***^).

## Results

### Anti-atherosclerosis effects of volatile oils of AMB and ACGD

**FIGURE 2:**
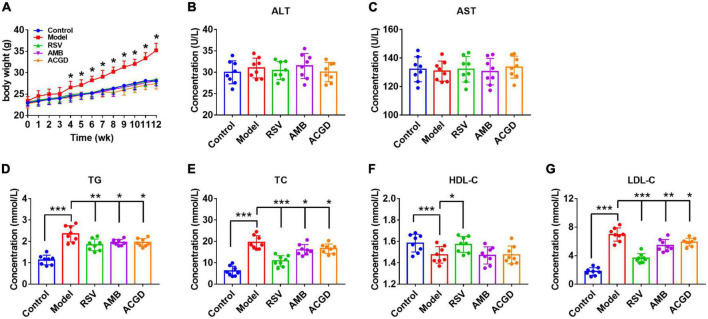
Effects of the volatile oils from *Allium macrostemon* Bge. (AMB) and *Allium chinense* G. Don (ACGD) on the body weight and serum levels of biochemical indexes in mice. **(A)** the level of body weight; the levels of serum alanine aminotransferase (ALT) **(B)**, aspartate aminotransferase (AST) **(C)**, triglyceride (TG) **(D)**, total cholesterol (TC) **(E)**, high-density lipoprotein cholesterol (HDL-C) **(F),** and low-density lipoprotein cholesterol (LDL-C) **(G)**. Data were expressed as mean ± SD (*n* = 8). (*p < 0.05, **p < 0.01, ***p < 0.001).

Compared with control groups, high fat and high cholesterol diets brought marked increase of body weight, TG, TC, and LDL-C levels, and reduction of HDL-C levels in model mice serum (Figure 2). Meanwhile, pretreatment with RSV, the volatile oils of AMB and ACGD all could obviously attenuate the elevation of body, weight, TG, TC, and LDL-C levels, suggesting their significant lipid lowering effects. However, almost no changes of HDL-C levels were observed after treatment the model mice with the volatile oils of AMB or ACGD. In addition, the results involving in ALT and AST levels indicated that the volatile oils of AMB and ACGD were without hepatotoxicity (Figures 2B, C), which kept in line with previous study (25).

**FIGURE 3:**
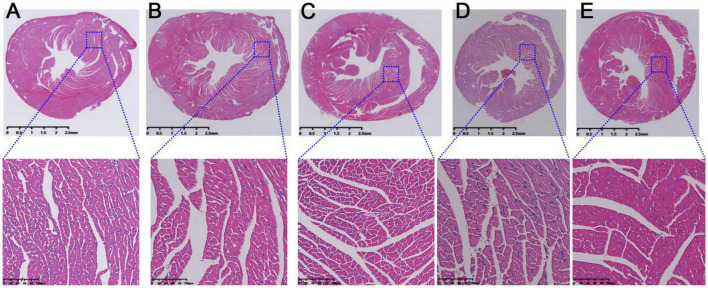
The hematoxylin-eosin (HE) staining results of heart tissues in mice from control group **(A)**, model group **(B)**, rosuvastatin calcium tablets (RSV) group **(C)**, *Allium macrostemon* Bge. (AMB) group **(D),** and *Allium chinense* G. Don (ACGD) group **(E)**.

During the course of atherosclerosis, pathological changes would appear in heart tissue when the myocardium was damaged. As shown in Figure 3A, the mice myocardium in control group was uniformly stained and the cardiomyocytes were neatly arranged. In addition, their morphology and structure were complete, and no cell swelling or necrosis was found. In contrast, the arrangement of myocardial cells was loose and the gap between muscle fibers was large in model mice (Figure 3B). Meanwhile, some nuclei were blurred or even disappeared, and obvious defects and blank areas are visible (Figure 3B). After the mice were treated with RSV, AMB, or ACGD, the morphology and arrangement of cardiomyocytes had significantly improved as shown in Figures 3C–E, respectively.

**FIGURE 4:**
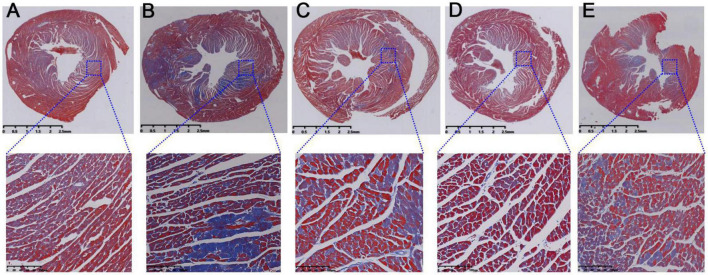
The Masson staining results of mice heart tissues in control group **(A)**, model group **(B)**, rosuvastatin calcium tablets (RSV) group **(C)**, *Allium macrostemon* Bge. (AMB) group **(D),** and *Allium chinense* G. Don (ACGD) group **(E)**.

**FIGURE 5:**
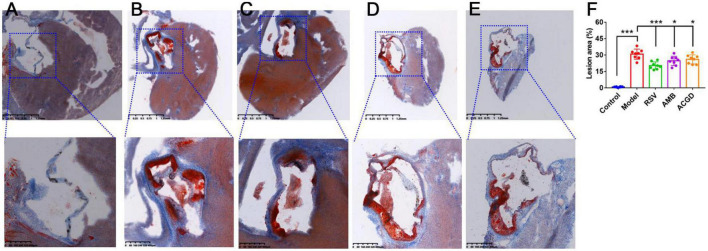
The Oil red O staining results of aortic valves in mice from control group **(A)**, model group **(B)**, rosuvastatin calcium tablets (RSV) group **(C)**, *Allium macrostemon* Bge. (AMB) group **(D),** and *Allium chinense* G. Don (ACGD) group **(E)**. And the comparison of atherosclerotic plaque **(F)**. Data were presented as mean ± SD (*n* = 8). (*p < 0.05, ***p < 0.001).

Further, Masson staining results were used to investigate the injury of myocardial fibrosis. Compared with the normal mice (Figure 4A), the myocardial fibrosis in model mice was obviously aggravated (Figure 4B). And this fibrosis was significantly relieved after intragastric administration of RSV (Figure 4C), AMB (Figure 4D), and ACGD (Figure 4E), respectively. Moreover, the results of oil red O staining for aortic valve showed that significant atherosclerotic plaque was deposited in the aortic intima in model mice (Figure 5A), and almost no plaques were found in normal mice (Figure 5B). Meanwhile, several fibrous caps were also observed. The lesion areas of plaques in RSV group (Figure 5C), AMB group (Figure 5D), and ACGD group (Figure 5E) were all significantly reduced (Figure 5F), and the treatment groups significantly prevented the formation of fiber caps, indicating their therapeutic effects of anti-atherosclerotic plaque formation. Taken together, these findings suggested that the volatile oils derived from AMB and ACGD both exhibited remarkable anti-atherosclerotic effects.

### Identification and characterization of VOCs in AMB and ACGD bulbs

A total of 121 and 115 volatile organic compounds were identified and characterized in the volatile oils of AMB and ACGD bulbs by HS-SPME-GC/MS, respectively (Supplementary Table 1). The overlay analysis of total ion chromatograms (TIC) for QC samples demonstrated the high stability of the instrument (Supplementary Figure 1A). Coefficients of variation derived from empirical cumulative distribution function illustrated the obtained data have a good stability (Supplementary Figure 1B). Furthermore, the TIC analysis of VOCs in AMB and ACGD show the recorded data have several similarities in chemical profiles and significant differences in content levels (Supplementary Figures 2A, B). And the VOCs were classified to sulfur compounds, heterocyclic compounds, esters, aldehydes, phenols, alcohols, ketones, aromatics, hydrocarbons, acids, amines, and terpenoids (Supplementary Figures 2C, D). Obviously, sulfur compounds contributed the most numbers, followed by heterocyclic compounds and esters, which accounted for over 70 VOCs both in AMB and ACGD. As described previously, the abundant sulfur compounds could well explain the garlic flavor of AMB and ACGD bulbs (26, 32). Meanwhile, this study systematically reported the holistic VOCs except sulfur compounds in AMB and ACGD for the first time.

### Relative-quantification of VOCs in AMB and ACGD bulbs

**FIGURE 6:**
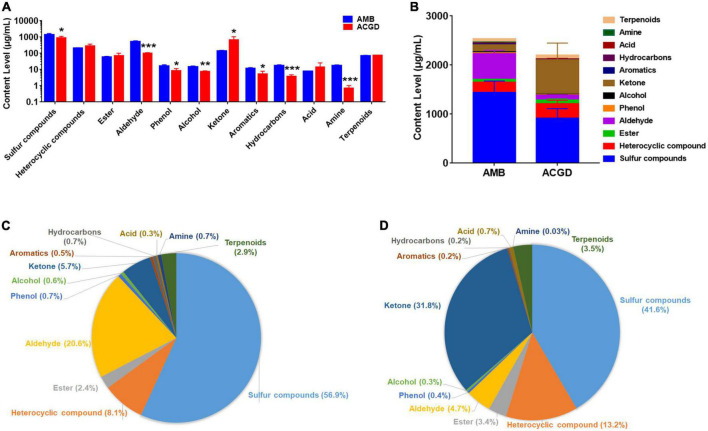
The comparison of content levels of holistic volatile organic compounds (VOCs) in *Allium macrostemon* Bge. (AMB) and *Allium chinense* G. Don (ACGD). Content comparison of multiple types of VOCs **(A)**. The total content comparison of holistic VOCs **(B)**. The content proportion of multiple types of VOCs in AMB **(C)** and ACGD **(D)**. Error bars represented the standards deviations of three determinations (*n* = 3) (*p < 0.05, **p < 0.01, ***p < 0.001).

The relative-contents of VOCs in AMB and ACGD were obtained based on the internal standard method as described previously (Supplementary Table 2). Obviously, total contents of sulfur compounds, aldehydes, phenols, alcohols, aromatics, hydrocarbons, and amines in ACGD were significantly lower than those in AMB (Figure 6A). In contrast, the contents of ketones were more abundant in ACGD (Figure 6A). Similarly, the total contents of VOCs in AMB were significantly higher than those in ACGD (Figure 6B). Further proportion analysis displayed that sulfur compounds, aldehydes, and heterocyclic compounds were the richest components and accounted for about 85.6% in AMB bulbs (Figure 6C). In addition, approximately 86.6% in ACGD bulbs were attributed to sulfur compounds, ketones, and heterocyclic compounds (Figure 6D). In these two vegetables, sulfur compounds were the main VOCs in AMB (56.9%) and ACGD (41.6%), and like to the therapeutic VOCs responsible for the lipid-lowering and anti-atherosclerotic effects.

### Identification of differential VOCs between AMB and ACGD samples

**FIGURE 7:**
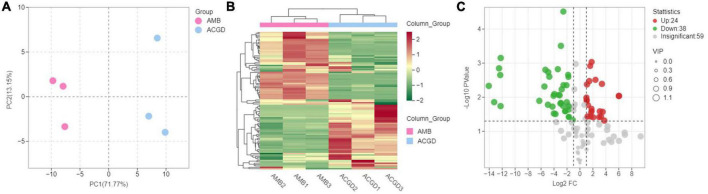
The principal component analysis (PCA) between *Allium macrostemon* Bge. (AMB) and *Allium chinense* G. Don (ACGD) samples by HS-SPME-GC/MS **(A)**; volatile organic compounds (VOCs) accumulation pattern of AMB and ACGD **(B)**; volcano plots of differentially VOCs between AMB and ACGD **(C)**; the green dots represent down-accumulated compounds, and the red dots represent up-accumulated volatiles between these two vegetables.

Principal component analysis (PCA) was a well-accepted method to investigate the differences between groups or variation within the group (31). The PCA results displayed the first principal component (PC1) and second principal component (PC2) could explain 71.77 and 13.15% of the total variances (Figure 7A). The PC1 scores plot showed that the instrument status was stable due to the PC1 values of experimental and QC samples within ± 2 × standard deviation (Supplementary Figure 3A). The significant separated samples indicated that there were several marked differences between the accumulation of VOCs in AMB and ACGD.

Hierarchal clustering analysis (HCA) of the VOCs in AMB and ACGD was carried out, and the results are shown in the heatmap (Figure 7B). Two main clusters were detected in the pattern of the VOCs accumulation. Over half of the VOCs significantly decreased in ACGD bulbs, while the remaining VOCs accumulated significantly in ACGD bulbs. In addition, as shown in Supplementary Figure 3B, the higher the correlation coefficient of intra-group samples relative to inter-group samples, the more reliable the differential VOCs obtained. These findings suggested that differentially accumulated VOCs are very important for elucidating the effective components of anti-atherosclerosis in AMB and ACGD.

Orthogonal partial least squares discriminant analysis (OPLS-DA) with T score of 76.00% and orthogonal T score of 6.23% (Supplementary Figure 4A) was used to screen the variances through deleting unrelated variances. The R^2^X (0.823) and Q^2^ (0.982) derived from OPLS-DA were both over 0.8, indicating OPLS-DA model did not overfit and had a high separating capacity (Supplementary Figure 4B). Further, the VOC variances significantly varied in AMB and ACGD bulbs based on obtained variable influence on projection (VIP) plot (Supplementary Figure 4C). According to the following principles: VIP ≥ 1, p-value < 0.05, fold change ≥ 2, or fold change ≤ 0.5, the differential VOCs in AMB and ACGD bulbs were determined. Compared with AMB, 38 VOCs were significantly decreased and 24 VOCs were up-regulated in ACGD group (Figure 7C and Table 1). These differentially expressed VOCs could be key factors for anti-atherosclerosis effects. Therefore, we further focused on the quantitative analysis of these significantly upregulated or down-regulated VOCs.

**TABLE 1:** The details of 62 differential volatile organic compounds (VOCs) between the volatile oils derived from the fresh bulbs of *Allium macrostemon* Bge. (AMB) and *Allium chinense* G. Don (ACGD).

Index	Compounds	Class	VIP	*P*-value	FDR	Fold change	Log_2_FC	Type
KMW0045	Dimethyl disulfide	Sulfur compounds	1.1004	0.00309	0.0249	5.2758	2.3994	Up
NMW0777	2-allyl methyl disulfide	Sulfur compounds	1.1269	0.04843	0.0814	13.6088	3.7665	Up
D384	Allyl methyl trisulfide	Sulfur compounds	1.1277	0.02677	0.0549	11.3935	3.5101	Up
XMW0921	1-thien-2-ylpentan-1-one	Sulfur compounds	1.1382	0.00917	0.0370	64.9006	6.0202	Up
KMW0401	Dimethyl tetrasulfane	Sulfur compounds	1.0495	0.01695	0.0456	2.3912	1.2577	Up
XMW1414	5-amino-2-ethyl-2,4-dihydro-4-methyl-3*H*-1,2,4-triazole-3-thione	Sulfur compounds	1.1242	0.00335	0.0253	2.7236	1.4455	Up
XMW3428	3,4-dimethylthiophene	Sulfur compounds	1.1393	0.00879	0.0370	0.1144	-3.1284	Down
XMW2185	2-methylthiolane 1,1-dioxide	Sulfur compounds	1.1386	0.02995	0.0594	0.0615	-4.0226	Down
KMW0352	Methyl 2-methyl-3-furyl disulfide	Sulfur compounds	1.1128	0.02555	0.0542	0.2107	-2.2470	Down
D33	1-(2,5-dimethyl-3-thienyl) ethanone	Sulfur compounds	1.1353	0.01883	0.0459	0.0531	-4.2354	Down
XMW0497	2,5-thiophenedicarboxaldehyde	Sulfur compounds	1.1338	0.01275	0.0439	0.0130	-6.2686	Down
XMW3737	5-acetyl-2-methyl-3-thiophenecarbaldehyde	Sulfur compounds	1.1268	0.01788	0.0459	0.1637	-2.6105	Down
WMW0071	Dipropyl trisulfane	Sulfur compounds	1.0774	0.04652	0.0793	0.4731	-1.0798	Down
GMW0100	(*E*)-propenyl propyl trisulfide	Sulfur compounds	1.1399	0.00184	0.0220	0.0338	-4.8883	Down
D268	Diallyl tetrasulfide	Sulfur compounds	1.0613	0.00255	0.0220	0.2917	-1.7774	Down
KMW0600	Bis(2-methyl-3-furyl)disulfide	Sulfur compounds	1.1365	0.00587	0.0296	0.1423	-2.8126	Down
XMW1702	3-ethoxy-2-(2-thienylmethylsulfonyl)-propennitrile	Sulfur compounds	1.1459	0.00465	0.0267	0.0001	-14.0663	Down
XMW1685	3,4-methylpropylsuccinimide	Heterocyclic compounds	1.1300	0.00421	0.0267	2.1242	1.0869	Up
KMW0380	2-isobutyl-3-methoxypyrazine	Heterocyclic compounds	1.1382	0.00917	0.0370	64.9006	6.0202	Up
XMW0163	2-hexylfuran	Heterocyclic compounds	1.1346	0.02600	0.0542	8.3865	3.0681	Up
XMW0435	2,4-dimethylfuran	Heterocyclic compounds	1.1376	0.00485	0.0267	0.0224	-5.4778	Down
NMW0017	*N*-methyl pyrrolidone	Heterocyclic compounds	1.1174	0.02572	0.0542	0.2048	-2.2879	Down
KMW0286	Isopropyl methoxy pyrazine	Heterocyclic compounds	1.0586	0.03730	0.0681	0.3099	-1.6902	Down
XMW0740	3-methyl-pyrrolo(2,3-b) pyrazine	Heterocyclic compounds	1.1443	0.00031	0.0188	0.1306	-2.9366	Down
XMW1877	4-(1-methyl propylamino)-pyrido-[3,2-c]-pyridazine	Heterocyclic compounds	1.0682	0.00851	0.0370	0.1015	-3.3007	Down
NMW0335	Quinolone	Heterocyclic compounds	1.1457	0.00070	0.0211	0.0002	-12.2266	Down
XMW1703	(2,3-dihydro-5-benzofuryl)-(4-morpholyl)-methanone	Heterocyclic compounds	1.1223	0.00003	0.0037	0.1698	-2.5582	Down
XMW0205	Benzimidazo[2,1-a]isoquinoline	Heterocyclic compounds	1.1238	0.01400	0.0446	0.2446	-2.0315	Down
XMW0955	4-formyl-3,5-dimethyl-1H-pyrrole-2-carbonitrile	Heterocyclic compounds	1.0908	0.00219	0.0220	0.2836	-1.8183	Down
NMW0424	7-acetoxy-4-methylcoumarin	Heterocyclic compounds	1.1459	0.00142	0.0211	0.0002	-12.4820	Down
XMW1085	Butyl 2-butenoate	Ester	1.0316	0.01226	0.0436	2.0258	1.0185	Up
XMW3765	Vinyl 2-ethylhexanoate	Ester	1.1233	0.03698	0.0681	9.4804	3.2449	Up
XMW3158	1-propanoyloxypropyl propanoate	Ester	1.0404	0.02547	0.0542	3.5270	1.8184	Up
XMW0506	D-alanine-*N*-propargyloxy carbonyl-propargyl ester	Ester	1.0952	0.00092	0.0211	3.4929	1.8044	Up
XMW1848	d-proline-*N*-methoxycarbonyl-methyl ester	Ester	1.1399	0.00806	0.0370	0.0651	-3.9418	Down
XMW1827	Phthalic acid-butyl hex-2-yn-4-yl ester	Ester	1.1214	0.02312	0.0528	0.2937	-1.7677	Down
KMW0066	Hexanal	Aldehyde	1.0037	0.01217	0.0436	2.1258	1.0880	Up
KMW0095	2-hexenal	Aldehyde	1.0870	0.00121	0.0211	2.9795	1.5751	Up
GMW0099	(*E*)-2-heptenal	Aldehyde	1.0864	0.03666	0.0681	2.2394	1.1631	Up
KMW0253	2-octenal	Aldehyde	1.0757	0.02174	0.0506	3.4541	1.7883	Up
WMW0047	5-ethylcyclopentene-1-carbaldehyde	Aldehyde	1.0971	0.03882	0.0681	4.1843	2.0650	Up
KMW0212	Phenylacetaldehyde	Aldehyde	1.0810	0.02798	0.0564	3.2920	1.7189	Up
KMW0407	(2*E*,4*E*)-2,4-non-adienal	Aldehyde	1.1139	0.03837	0.0681	7.8444	2.9717	Up
XMW1375	2-methyl-2-pentenal	Aldehyde	1.1430	0.01658	0.0456	0.0327	-4.9354	Down
XMW0483	2-formyl-1H-pyrrole	Aldehyde	1.1458	0.01393	0.0446	0.0001	-13.2311	Down
XMW1714	4-(1-phenyl-2-propenyloxy)-benzaldehyde	Aldehyde	1.1349	0.01534	0.0456	0.1249	-3.0015	Down
XMW0507	Olivetol	Phenol	1.1365	0.00405	0.0267	10.5205	3.3951	Up
XMW3151	3,4,5-trimethylphenol	Phenol	1.1457	0.00223	0.0220	0.0002	-12.2976	Down
XMW1108	2,4,6-tri(propan-2-yl)phenol	Phenol	1.1084	0.01460	0.0453	0.1303	-2.9403	Down
KMW0523	1-undecanol	Alcohol	1.1372	0.03104	0.0606	0.0731	-3.7741	Down
XMW1077	2,2’-(1,3-dioxolane-2,2-diyl) diethanol	Alcohol	1.1444	0.01819	0.0459	0.0002	-12.2468	Down
KMW0674	(*E*)-2-dodecen-1-ol	Alcohol	1.1278	0.01852	0.0459	0.3007	-1.7337	Down
NMW0339	Tryptophol	Alcohol	1.1432	0.00249	0.0220	0.0379	-4.7224	Down
XMW0637	2,2,3-trimethylcyclobutanone	Ketone	1.1079	0.03682	0.0681	3.1162	1.6398	Up
XMW0973	Methyl(2,4,5-trimethyl-1H-pyrrol-3-yl) ketone	Ketone	1.0240	0.01306	0.0439	2.0477	1.0340	Up
XMW0508	2,5-dihydroxy-6-propan-2-ylcyclohepta-2,4,6-trien-1-one	Ketone	1.0655	0.01018	0.0397	2.0135	1.0097	Up
XMW0581	1,2-dimethoxybenzene	Aromatics	1.1454	0.00157	0.0211	0.0249	-5.3260	Down
KMW0727	Heneicosane	Hydrocarbons	1.1236	0.00150	0.0211	0.0939	-3.4131	Down
ZMW0001	Tricosane	Hydrocarbons	1.0978	0.00444	0.0267	0.2387	-2.0667	Down
WMW0132	Pentadecanoic acid	Acid	1.1081	0.00442	0.0267	0.1712	-2.5460	Down
XMW0124	3-aminophenylacetylene	Amine	1.1411	0.00904	0.0370	0.0599	-4.0602	Down
XMW2159	4-(2-methoxypropan-2-yl)-1-methylcyclohex-1-ene	Terpenoids	1.0452	0.03814	0.0681	0.2981	-1.7463	Down

**FIGURE 8:**
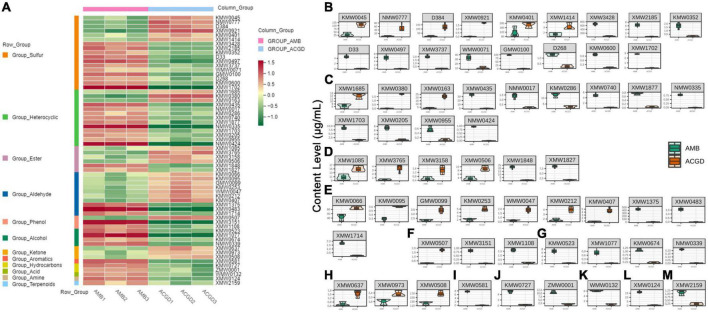
The accumulation pattern of 62 differential volatile organic compounds (VOCs) between *Allium macrostemon* Bge. (AMB) and *Allium chinense* G. Don (ACGD) **(A)**, red color and green color represent up-accumulated and down-accumulated compounds, respectively; comparison of relative amounts of 62 differential VOCs including 17 sulfur compounds **(B)**, 13 heterocyclic compounds **(C)**, 6 esters **(D)**, 10 aldehydes **(E)**, 3 phenols **(F)**, 4 alcohols **(G)**, 3 ketones **(H)**, 1 aromatic **(I)**, 2 hydrocarbon **(J)**, 1 acid **(K)**, 1 amine **(L)**, and 1 terpenoid **(M)** in AMB and ACGD samples.

To better understand the main VOCs changes between AMB and ACGD bulbs, a visual heat-map including 62 differentially expressed VOCs were displayed in Figure 8A. Among them, sulfur compounds (17) were identified as the main differential VOCs, followed by heterocyclic compounds (13), aldehydes (10), and esters (6). Sulfur compounds were responsible for garlic flavor, whereas aldehydes, esters, ketones, and terpenoids contributed more to a wide spectrum of aromas. Furthermore, quantitative results showed that six sulfur compounds obviously were up-regulated in ACGD bulbs, especially the main VOCs including dimethyl disulfide (KMW0045), 2-allyl methyl disulfide (NMW0777), allyl methyl trisulfide (D384), dimethyl tetrasulfane (KMW0401) (Figure 8B). Similarly, 3,4-dimethylthiophene (XMW3428), dipropyl trisulfane (WMW0071) and (*E*)-propenyl propyl trisulfide (GMW0100) were more abundant in AMB than ACGD bulbs (Figure 8B).

However, few studies revealed the differences of VOCs except sulfur compounds in these two edible vegetables. In the present study, we have confirmed that the relative contents of 3,4-methylpropylsuccinimide (XMW1685) and 2-hexylfuran (XMW0163) were higher in ACGD, whereas 2,4-dimethylfuran (XMW0435), isopropyl methoxy pyrazine (KMW0286), (2,3-dihydro-5-benzofuryl)-(4-morpholyl)-methanone (XMW1703) and benzimidazole [2,1-a]isoquinoline (XMW0205) were more abundant in AMB (Figure 8C). Likewise, esters including butyl 2-butenoate (XMW1085) and d-proline-N-methoxycarbonyl-methyl ester (XMW1848) (Figure 8D), aldehydes including hexanal (KMW0066) and 2-methyl-2-pentenal (XMW1375) (Figure 8E), ketone containing 2,5-dihydroxy-6-propan-2-ylcyclohepta-2,4,6-trien-1-one (XMW0508) (Figure 8H), hydrocarbon including tricosane (ZMW0001) (Figure 8J) and amine including 3-aminophenylacetylene (XMW0124) (Figure 8L) were also identified as the major contributors to the differences between AMB and ACGD samples. Except these main VOCs (over 10 μg/g), several minor differentially VOCs with less than 10 μg/g were also presented in Figures 8B–M. These findings indicated that the abundant VOCs differed during the development and ripening of AMB and ACGD bulbs.

## Discussion

A major finding in this study was that the volatile oils derived from AMB and ACGD both showed similar and close lipid lowering effect (Figure 2) and anti-atherosclerosis activity (Figures 3–5), which provided strong evidences for their traditional efficacy for the prevention and treatment of chest stuffiness and pains. The lipid lowering effects of AMB and ACGD toward TC and LDL-C levels were significantly weaker than those of rosuvastatin, especially HDL-C levels (Figure 2). Traditionally, because statins have similar chemical structure with HMG-CoA and their affinity binding to HMG-CoA reductase is thousands of times higher than HMG-CoA, these statins including rosuvastatin could competitively inhibit HMG-CoA reductase, further block the metabolic pathway of mevalonate and reduce the synthesis of cholesterol in cells (33, 34). They demonstrated the strongest effect on lowering LDL-C, followed by TC, with little effect on lowering TG, and HDL-C slightly increased (33, 34). These obvious effects on regulating blood lipid and treating cardiovascular diseases have been widely recognized for over 40 years (33, 34). With the advanced researches, the protective effects of statins were additionally attributed to their anti-inflammatory, immunomodulatory functions, antioxidative and anti-thrombotic activities rather than lipid-lowering abilities alone (34).

In this study, the potential benefits for cardiovascular diseases of AMB and ACGD volatile oils could be compared with the efficacy and functions of garlic oil (a commercial product) due to several common sulfur compounds including diallyl disulfide, propenyl propyl disulfide, 2-allyl methyl disulfide, etc. (35, 36). Numerous clinical trials have proved garlic oil could result in a reduction in total cholesterol. Other important benefits had been observed in garlic’s ability to reduce the ability of platelets to aggregate, inhibit enzymes involved in lipid synthesis, and inhibit angiotensin-converting enzyme (27, 35). For example, garlic oil could also inhibit human HMG-CoA reductase and squalene monooxygenase just like statins (37, 38). In addition, the garlic’s constituents are likely to work *via* the inhibition of calcium mobilization or modulating the cyclooxygenase activity to exert platelet aggregation (39, 40). Further, these volatile oils also showed blood coagulation, fibrinolysis and circulatory effects, and effects on endogenous antioxidant defenses (35). An obvious limitation was that we did not investigate the regulatory effects of the volatile oil from AMB and ACGD toward the key enzymes mentioned above. This also led to the lack of direct evidence to compare the VOC profiles and pharmacological effects of these two herbs with garlic oil.

The sulfur-containing compounds in AMB and ACGD volatile oils were like the main contributors to lipid lowering and anti-atherosclerosis effects. This was because several naturally occurring sulfur-containing compounds (*Allium* family) including diallyl disulfide (GMW0113), diallyl trisulfide (GMW0108) and methyl propyl trisulfide (GMW0115) could improve the cardiac damages and ameliorate cardiac dysfunction through the roles of AMPK-mediated AKT/GSK-3β/HIF-1α activation, PI3K/Akt-SREBP2 pathway, and eNOS-Nrf2-Tfam pathway (25, 27, 41–44). And diallyl disulfide and its mono-*S*-oxide (allicin) were thought to be the most active sulfur-containing components to influence plasma cholesterol and atherosclerosis (45, 46). The safety of volatile oils derived from herbs or foods is worthy of attention (47). Our results indicated that no significant adverse effects (ALT and AST levels in Figures 2B, C) were observed which kept in line with previous reports (25, 27, 41). However, garlic oils in large quantities actually have significant side effects like anemia or allergic manifestations (48). Except individual sulfur-containing compounds, other main VOCs like 5-methyl-2-octylfuran-3-one (XMW3286), 2-methyl-2-pentenal (XMW1375) were reported with anti-microbial, anti-oxidant and anti-inflammatory activity (49, 50). For most VOCs in AMB and ACGD, there were a lack of researches on their pharmacological effects, especially anti-atherosclerosis activity. This also made it impossible to judge the contribution of some VOCs to the holistic effects. Therefore, much more work is needed to further investigate the chemical profiles and pharmacology of sulfur-containing VOCs in these plants.

In addition, sulfur-containing compounds also contributed more to strong odors of AMB and ACGD samples. Traditionally, when the bulb tissues were chopped or homogenated, several thiosulfinates and propanethial-*S*-oxide were the initial chemical compounds in *Allium* species (32, 51). After heating, these thiosulfinates decomposed immediately to additional sulfur compounds including diallyl, methyl allyl, and diethyl mono-, di-, tri-, tetra-sulfides, which were emitted as the major aroma compounds (26, 32). In this study, several thiophene compounds including 2-propylbenzo[b]thiophene (XMW2525), 2-hexylthiophene (XMW1061) and 2-thienylamide (XMW3292) were detected in volatile oils of AMB and ACGD (Supplementary Table 1), of which 3,4-dimethylthiophene (XMW3428) and 1-(2,5-dimethyl-3-thienyl)ethanone (D33) were considered as the thermal decomposition products from dialkyl disulfides (26, 52). However, individual sulfur-containing compounds including dimethyl trisulfide and methyl propyl disulfide were not detected in AMB or ACGD oils (16, 17). This may be attributed to their different producing areas, different harvest time and enzymolysis degree of extraction process. Obviously, it is necessary to perform a comparative qualitative and quantitative analysis of VOCs in AMB and ACGD from multiple producing areas and different growth stages.

Furthermore, identified or annotated VOCs in AMB and ACGD whose moieties included CH, CHO, CHON, or CHN atoms were categorized as heterocyclic compounds, esters, aldehydes, phenols, alcohols, or ketones (Supplementary Figures 2C, D). This was the first time to characterize the holistic VOCs in AMB and ACGD except sulfur-containing compounds (Supplementary Table 1). Several VOCs including alkanes, alcohols, aldehydes and esters were also detected in garlic oil or other *Allium* plants (53, 54). These esters, aldehydes, alcohols, and ketones probably contributed to the aromas of AMB and ACGD oils (29). This was because the precursors of many esters were fatty acids and amino acids, while aldehydes were derived from fatty acid oxidation or amino acid metabolism (55). Obviously, the content levels of these VOCs including 2,4-dimethylfuran (XMW0435), butyl 2-butenoate (XMW1085), 2-methyl-2-pentenal (XMW1375), 2,5-dihydroxy-6-propan-2-ylcyclohepta-2,4,6-trien-1-one (XMW0508), tricosane (ZMW0001), and 3-aminophenylacetylene (XMW0124) were significantly different between the volatile oils of AMB and ACGD (Figure 8). Their functions and biosynthetic pathways remained largely limited. And in-depth researches of these VOCs need to be carried out in future.

Moreover, *Allium* vegetables could produce the most abundant sulfur compounds, and other various structural types of VOCs (53, 56). This also put forward higher requirements for the selection of internal standard (IS) due to the utmost importance of IS for non-targeted metabolite profiling (57). Traditionally, due to the variety species of VOCs, one IS cannot well reflect all categorization of VOCs and is vulnerable to interference from the matrix (58). Several common volatile isotope ISs which could be commercially obtained are purchased and prepared to mixed solutions for further analysis. The most stable isotope IS in tested samples is selected as the appropriate IS for analysis (58). In the present study, a total of 16 isotope ISs including ester, aldehyde, phenol, alcohol, ketone, aromatics, hydrocarbons, and heterocyclic compounds with different physicochemical properties including retention times, retention index (RI), match factor was performed for the holistic VOCs analysis in AMB, ACGD, and QC samples (Supplementary Figure 5). The obtained results with coefficient of variation (CV) values were used to evaluate whether the VOCs participated in the cross contribution and whether the RI values of each IS was reproducible (58). Finally, [2H8]-acetophenone was selected as the appropriate IS with CV values of 11% in AMB, 12% in ACGD, and 18% in QC samples (Supplementary Table 3). In addition, according to the value of partition coefficient and the solubility of [2H8]-acetophenone, methanol was used as the solvent.

## Conclusion

The volatile oils of AMB and ACGD were first proved to have remarkable lipid-lowering (TG, TC, LDL-C) and anti-atherosclerosis effects (atherosclerotic plaque and fiber cap). Furthermore, a total of 121 VOCs were annotated or tentatively identified in the holistic profile of AMB and ACGD volatile oils based on retention time, retention index and match factor by HS-SPME-GC/MS. In addition, internal standards-mediated quantitative method was used to perform their relative content levels. Moreover, 62 differential expressed VOCs were screened between AMB and ACGD bulbs based on PCA and OPLS-DA approaches. Taken together, this study provided some scientific information about the lipid-lowering and anti-atherosclerosis functions of AMB and ACGD volatile oils as well as their holistic VOCs.

## Data availability statement

The original contributions presented in this study are included in this article/Supplementary material, further inquiries can be directed to the corresponding author.

## Ethics statement

The animal study was reviewed and approved by Laboratory Animal Ethics Committee of Zhengzhou University.

## Author contributions

ZQ and JY: conceptualization, methodology, investigation, data curation, formal analysis, and writing – original draft. SD, YL, XL, and HX: methodology, investigation, formal analysis, and data curation. ZY, XZ, and XY: conceptualization, supervision, and writing – review and editing. HX, ZQ, and JY: funding acquisition, project administration, and writing – review and editing. All authors have read and agreed with the manuscript.

## Footnotes

1
https://cloud.metware.cn

## References

[1] Yao Z Qin Z Dai Y Yao X. Phytochemistry and pharmacology of Allii Macrostemonis *Bulbus*, a traditional Chinese medicine. *Chin J Nat Med.* (2016) 14:481–98. 10.1016/S1875-5364(16)30058-9 27507199

[2] Chen S Wei C Gao P Kong H Jia Z Hu C Effect of Allium macrostemon on a rat model of depression studied by using plasma lipid and acylcarnitine profiles from liquid chromatography/mass spectrometry. *J Pharm Biomed Anal.* (2014) 89:122–9. 10.1016/j.jpba.2013.10.045 24284228

[3] Kyung K. Antimicrobial properties of allium species. *Curr Opin Biotechnol.* (2012) 23:142–7. 10.1016/j.copbio.2011.08.004 21903379

[4] Li F Xu Q Zheng T Huang F Han L. Metabonomic analysis of *Allium macrostemon* Bunge as a treatment for acute myocardial ischemia in rats. *J Pharm Biomed Anal.* (2014) 88:225–34. 10.1016/j.jpba.2013.09.002 24080525

[5] Liu W Xiong X Yang X Chu F Liu H. The effect of Chinese herbal medicine gualouxiebaibanxia decoction for the treatment of angina pectoris: a systematic review. *Evid Based Complementary Altern Med.* (2016) 2016:8565907. 10.1155/2016/8565907 27777598 PMC5061958

[6] National Health Commission. *The List of Edible Traditional Chinese Medicines (Exposure Draft). Official Correspondence No. 36, the Office of the National Health Commission of the People’s Republic of China.* Beijing: National Health Commission (2021).

[7] Choi H Giussani L Jang C Oh B Cota-Sánchez J. Systematics of disjunct northeastern Asian and northern North American *Allium* (Amaryllidaceae). *Botany.* (2012) 90:491–508. 10.1139/b2012-031

[8] Shahrajabian M Sun W Cheng Q. Chinese onion (*Allium chinense*), an evergreen vegetable: a brief review. *Polish J Agron.* (2020) 42:40–5. 10.26114/pja.iung.426.2020.42.05

[9] Zhang W Yu Y Yan L Li C Han J Qin Z Discovery of cardio-protective constituents of Gualou Xiebai decoction, a classical traditional Chinese medicinal formula. *Phytomedicine.* (2019) 54:318–27. 10.1016/j.phymed.2018.04.047 30060904

[10] Qin Z Dai Y Yao Z He L Wang Q Geng J Study on chemical profiles and metabolites of Allii Macrostemonis *Bulbus* as well as its representative steroidal saponins in rats by ultra-performance liquid chromatography coupled with quadrupole time-of-flight tandem mass spectrometry. *Food Chem.* (2016) 192:499–515. 10.1016/j.foodchem.2015.07.040 26304378

[11] Duan S Li X Yao Z Zhang X Yao X Yang J Visual authentication of steroidal saponins in *Allium macrostemon* Bge. and *Allium chinense* G. Don using MALDI-TOF imaging mass spectrometry and their structure activity relationship. *Arab J Chem.* (2022) 15:104138.

[12] Yang Y Huang C Su X Zhu J Chen X Fu Y Deciphering the multicomponent synergy mechanism from a systems pharmacology perspective: application to Gualou Xiebai decoction for coronary heart disease. *J Funct Foods.* (2018) 47:143–55. 10.1016/j.jff.2018.02.030

[13] Qin Z Lin P Dai Y Yao Z Wang L Yao X Quantification and semi-quantification of multiple representative components for the holistic quality control of Allii Macrostemonis *Bulbus* by ultra high performance liquid chromatography with quadrupole time-of-flight tandem mass spectrometry. *J Sep Sci.* (2016) 39:1834–41. 10.1002/jssc.201501368 26991139

[14] Qin Z Lin P Yao Z Chen Z Yu Y Dai Y Diagnostic ion-oriented identification and simultaneous quantification of chemical components in *Allium chinense* G. *Don. J Sep Sci.* (2018) 41:4253–71. 10.1002/jssc.201800476 30267555

[15] Lin P Qin Z Yao Z Wang L Zhang W Yu Y Metabolites profile of Gualou Xiebai Baijiu decoction (a classical traditional Chinese medicine prescription) in rats by ultra-performance liquid chromatography coupled with quadrupole time-of-flight tandem mass spectrometry. *J Chromatogr B.* (2018) 1085:72–88. 10.1016/j.jchromb.2018.04.001 29635208

[16] Liu X Lu X Liu Q Liu Z. Evaluation of insecticidal activity of the essential oil of *Allium chinense* G. Don and its major constituents against *Liposcelis bostrychophila* Badonnel. *J Asia Pac Entomol.* (2014) 17:853–6. 10.1016/j.aspen.2014.08.007

[17] Liu X Liu Q Zhou L Liu Z. Evaluation of larvicidal activity of the essential oil of *Allium macrostemon* Bunge and its selected major constituent compounds against *Aedes albopictus* (Diptera: Culicidae). *Parasit Vectors.* (2014) 7:184. 10.1186/1756-3305-7-184 24731297 PMC3996138

[18] Pino J Fuentes V Correa M. Volatile constituents of Chinese Chive (*Allium tuberosum* Rottl. ex Sprengel) and Rakkyo (*Allium chinense* G. Don). *J Agric Food Chem.* (2001) 49:1328–30. 10.1021/jf9907034 11312859

[19] Zhang Q Zhao Q Shen Y Zhao F Zhu Y. Allium vegetables, garlic supplements, and risk of cancer: a systematic review and meta-analysis. *Front Nutr.* (2022) 8:746944. 10.3389/fnut.2021.746944 35402472 PMC8985597

[20] Li F Zheng T Xu Q Huang F Liu X Han L. NMR spectroscopy and multivariate statistical analysis of metabonomic changes in *Allium macrostemon* Bunge extracts induced by different drying methods. *Anal Methods.* (2013) 5:6219. 10.1039/c3ay40626a

[21] Ohshima K Muraoka S Yasaka R Adachi S Tokuda M. First report of Scallion mosaic virus on wild Japanese garlic (*Allium macrostemon*) in Japan. *J Gen Plant Pathol.* (2016) 82:61–4. 10.1007/s10327-015-0636-5

[22] He Q Huang S Wu Y Zhang W Wang F Cao J Comparative study on the composition of free amino acids and derivatives in the two botanical origins of an edible Chinese herb “Xiebai”, i.e., *Allium chinense* G. Don and *Allium macrostemon* Bunge species. *Food Res Int.* (2018) 106:446–57. 10.1016/j.foodres.2018.01.007 29579946

[23] Lin Y Lin L Yeh H Chuang C Tseng S Yen Y. Antihyperlipidemic activity of *Allium chinense* bulbs. *J Food Drug Anal.* (2016) 24:516–26. 10.1016/j.jfda.2016.01.010 28911557 PMC9336657

[24] Han C Qi J Gao S Li C Ma Y Wang J Vasodilation effect of volatile oil from *Allium macrostemon* Bunge are mediated by PKA/NO pathway and its constituent dimethyl disulfide in isolated rat pulmonary arterials. *Fitoterapia.* (2017) 120:52–7. 10.1016/j.fitote.2017.05.007 28552597

[25] Bastaki M Aubanel M Cachet T Demyttenaere J Diop M Harman C Absence of adverse effects following the gavage administration of methyl propyl trisulfide to Sprague-Dawley rats for 90 days. *Food Chem Toxicol.* (2018) 120:544–51. 10.1016/j.fct.2018.07.056 30075317

[26] Jarvenpaa E Zhang Z Huopalahti R King J. Determination of fresh onion (*Allium cepa* L.) volatiles by solid phase microextraction combined with gas chromatography-mass spectrometry. *Z Lebensm Unters Forsch.* (1998) 207:39–43. 10.1007/s002170050292

[27] Asdaq S Challa O Alamri A Alsanie W Alhomrani M Asad M. The Potential benefits of using garlic oil and its active constituent, dially disulphide, in combination with carvedilol in ameliorating isoprenaline-induced cardiac damage in rats. *Front Pharmacol.* (2021) 12:739758. 10.3389/fphar.2021.739758 34646139 PMC8502798

[28] Li C Xin M Li L He X Yi P Tang Y Characterization of the aromatic profile of purple passion fruit (*Passiflora edulis* Sims) during ripening by HS-SPME-GC/MS and RNA sequencing. *Food Chem.* (2021) 355:129685. 10.1016/j.foodchem.2021.129685 33799248

[29] Kalogiouri N Manousi N Paraskevopoulou A Mourtzinos I Zachariadis G Rosenberg E. Headspace solid-phase microextraction followed by gas chromatography-mass spectrometry as a powerful analytical tool for the discrimination of truffle species according to their volatiles. *Front Nutr.* (2022) 9:856250. 10.3389/fnut.2022.856250 35558753 PMC9085510

[30] Wang H Hua J Jiang Y Yang Y Wang J Yuan H. Influence of fixation methods on the chestnut-like aroma of green tea and dynamics of key aroma substances. *Food Res Int.* (2020) 136:109479. 10.1016/j.foodres.2020.109479 32846562

[31] Tang Y Lv X Liu Y Cui D Wu Y. Metabonomics study in mice with learning and memory impairment on the intervention of essential oil extracted from *Cinnamomum camphora* Chvar. Borneol. *Front Pharmacol.* (2022) 13:770411. 10.3389/fphar.2022.770411 35359846 PMC8960444

[32] Rose P Whiteman M Moore P Zhu Y. Bioactive S-Alk(en)yl cysteine sulfoxide metabolites in the Genus *Allium*: the chemistry of potential therapeutic agents. *Nat Prod Rep.* (2005) 22:351–68. 10.1039/b417639c 16010345

[33] Rosenson R. Statins in atherosclerosis: lipid-lowering agents with antioxidant capabilities. *Atherosclerosis.* (2004) 173:1–12. 10.1016/S0021-9150(03)00239-9 15177118

[34] Parihar S Guler R Brombacher F. Statins: a viable candidate for host-directed therapy against infectious diseases. *Nat Rev Immunol.* (2019) 19:104–17. 10.1038/s41577-018-0094-3 30487528

[35] Rahman K Lowe G. Garlic and cardiovascular disease: a critical review. *J Nutr.* (2006) 136:736S–40S. 10.1093/jn/136.3.736S 16484553

[36] Qin W Huber K Popp M Bauer P Buettner A Sharapa C Quantification of Allyl Methyl Sulfide, Allyl methyl sulfoxide, and Allyl methyl sulfone in human milk and urine after ingestion of cooked and roasted garlic. *Front Nutr.* (2020) 7:565496. 10.3389/fnut.2020.565496 33072797 PMC7531236

[37] Gebhardt R. Multiple inhibitory effects of garlic extracts on cholesterol biosynthesis in hepatocytes. *Lipids.* (1993) 28:613–9. 10.1007/BF02536055 8394977

[38] Gupta N Porter T. Garlic and garlic derived compounds inhibit human squalene monooxygenase. *J Nutr.* (2001) 131:1662–7. 10.1093/jn/131.6.1662 11385050

[39] Qi R Liao F Inoue K Yatomi Y Sato K Ozaki Y. Inhibition by diallyl trisulfide, a garlic component, of intracellular Ca2+ mobilization without affecting inositol-1,4,5-triphosphate (IP3) formation in activated platelets. *Biochem Pharmacol.* (2000) 60:1475–83. 10.1016/S0006-2952(00)00467-6 11020449

[40] Chang H Yamato O Yamasaki M Maede Y. Modulatory influence of sodium 2-propenyl thiosulfate from garlic on cyclooxygenase activity in canine platelets: possible mechanism for the anti-aggregatory effect. *Prostaglandins Leukot Essent Fatty Acids.* (2005) 72:351–5. 10.1016/j.plefa.2005.01.003 15850716

[41] Huang Y Yao C Way C Lee K Tsai C Ou H Diallyl trisulfide and diallyl disulfide ameliorate cardiac dysfunction by suppressing apoptotic and enhancing survival pathways in experimental diabetic rats. *J Appl Phycol.* (2012) 114:402–10. 10.1152/japplphysiol.00672.2012 23139364

[42] Wu Y Li L Sun X Wang J Ma C Zhang Y Diallyl disulfide improves lipid metabolism by inhibiting PCSK9 expression and increasing LDL uptake via PI3K/Akt-SREBP2 pathway in HepG2 cells. *Nutr Metab Cardiovasc Dis.* (2021) 31:322–32. 10.1016/j.numecd.2020.08.012 33500108

[43] Khatua T Dinda A Putcha U Banerjee S. Diallyl disulfide ameliorates isoproterenol induced cardiac hypertrophy activating mitochondrial biogenesis via eNOS-Nrf2-Tfam pathway in rats. *Biochem Biophys Rep.* (2015) 5:77–88. 10.1016/j.bbrep.2015.11.008 28955809 PMC5600345

[44] El-Ashmawy N Khedr N Shaban M Al-Ashmawy G. Diallyl trisulfide modulated autophagy in isoproterenol induced acute myocardial infarction. *Clin Phytosci.* (2022) 8:20. 10.1186/s40816-022-00351-2

[45] Howard B Kritchevsky D. Phytochemicals and cardiovascular disease. A statement for healthcare professionals from the American Heart Association. *Circulation.* (1997) 95:2591–3. 10.1161/01.cir.95.11.2591 9184593

[46] Sendl A Schliack M Löser R Stanislaus F Wagner H. Inhibition of cholesterol synthesis in vitro by extracts and isolated compounds prepared from garlic and wild garlic. *Atherosclerosis.* (1992) 94:79–85. 10.1016/0021-9150(92)90190-R 1632861

[47] Chen W Luo H Zhong Z Wei J Wang Y. The safety of Chinese medicine: a systematic review of endogenous substances and exogenous residues. *Phytomedicine.* (2023) 108:154534. 10.1016/j.phymed.2022.154534 36371955

[48] Kritchevsky D. The effect of dietary garlic on the development of cardiovascular disease. *Trends Food Sci Technol.* (1991) 2:141–4. 10.1016/0924-2244(91)90658-6

[49] Shalaby E Nasr N Sherief S. An in vitro study of the antimicrobial and antioxidant efficacy of some nature essential oils. *J Med Plant Res.* (2011) 5:922–31. 10.5897/JMPR.9000072

[50] Sami R Elhakem A Alharbi M Almatrafi M Benajiba N Mohamed T In-vitro evaluation of the antioxidant and anti-inflammatory activity of volatile compounds and minerals in five different onion varieties. *Separations.* (2021) 8:57. 10.3390/separations8050057

[51] Shen C Xiao H Parkin K. In vitro stability and chemical reactivity of thiosulfinates. *J Agric Food Chem.* (2002) 50:2644–51. 10.1021/jf011013e 11958636

[52] Block E Putman D Zhao S. Allium chemistry: GC-MS analysis of thiosulfinates and related compounds from onion, leek, scallion, shallot, chive, and Chinese chive. *J Agric Food Chem.* (1992) 40:2431–8. 10.1021/jf00024a018

[53] Kusano M Kobayashi M Iizuka Y Fukushima A Saito K. Unbiased profiling of volatile organic compounds in the headspace of Allium plants using an in-tube extraction device. *BMC Res Notes.* (2016) 9:133. 10.1186/s13104-016-1942-5 26928722 PMC4772445

[54] Yusuf O Bewaji C. Evaluation of essential oils composition of methanolic *Allium sativum* extract on *Trypanosoma brucei* infected rats. *Res Pharm Biotech.* (2011) 3:17–21. 10.5897/RPB.9000012

[55] Li H Wu Q Liu Q Jin L Chen B Li C Volatile flavor compounds of *Pugionium cornutum* (L.) Gaertn. Before and after different dehydration treatments. *Front Nutr.* (2022) 9:884086. 10.3389/fnut.2022.884086 35586736 PMC9108931

[56] Khatua T Borkar R Mohammed S Dinda A Srinivas R Banerjee S. Novel sulfur metabolites of garlic attenuate cardiac hypertrophy and remodeling through induction of Na+/K+-ATPase expression. *Front Pharmacol.* (2017) 8:18. 10.3389/fphar.2017.00018 28194108 PMC5276815

[57] Houriet J Allard P Queiroz E Marcourt L Gaudry A Vallin L A mass spectrometry based metabolite profiling workflow for selecting abundant specific markers and their structurally related multi-component signatures in traditional chinese medicine multi-herb formulae. *Front Pharmacol.* (2020) 11:578346. 10.3389/fphar.2020.578346 33362543 PMC7756971

[58] Redestig H Fukushima A Stenlund H Moritz T Arita M Saito K Compensation for systematic cross-contribution improves normalization of mass spectrometry based metabolomics data. *Anal Chem.* (2009) 81:7974–80. 10.1021/ac901143w 19743813

