# Supplementary material for: Characterization of volatile organic compounds with anti-atherosclerosis effects in Allium macrostemon Bge. and Allium chinense G. Don by head space solid phase microextraction coupled with gas chromatography tandem mass spectrometry
Source: Front Nutr. 2023 Feb 1;10:996675. doi: 10.3389/fnut.2023.996675 (PMC9929146; doi:10.3389/fnut.2023.996675)
Supplement: Supplementary file 1 [file Data_Sheet_1.docx]

**Supporting information**

Characterization of volatile organic compounds with anti-atherosclerosis effects in *Allium macrostemon* Bge. and *Allium chinense* G. Don by head space solid phase microextraction coupled with gas chromatography tandem mass spectrometry

Zifei Qin ^1, 2, 3^, Shuyi Duan ^1^, Yuan Li ^1^, Xinqiang Li ^4^, Han Xing ^1, 2^, Zhihong Yao ^3^, Xiaojian Zhang ^1, 2^, Xinsheng Yao ^3^, Jing Yang ^1, 2^*

^1^ Department of Pharmacy, the First Affiliated Hospital of Zhengzhou University, Zhengzhou 450052, China;

^2^ Henan Applied & Translational Center of Precision Clinical Pharmacy, Zhengzhou 450052, China;

^3^ College of Pharmacy, Jinan University, Guangzhou 510632, China;

^4^ Department of Pathology, the First Affiliated Hospital of Zhengzhou University, Zhengzhou 450052, China;

*** Correspondence:**

jingyang_0101@163.com (Jing Yang)

**Table lists**

**Table S1** The detailed information of 121 volatile organic compounds in fresh bulbs of AMB and ACGD samples.

**Table S2** Content levels (μg/g) of VOCs in AMB and ACGD samples.

**Table S3** The detailed information of 16 internal standards in AMB and ACGD samples.

**Figure captions**

**Figure S1** The overlay total ion chromatograms (TICs) of QC samples (A) and coefficient of variation (CV) plot of multiple AMB and ACGD samples.

**Figure S2** Total ion chromatograms (TICs) of holistic VOCs in the volatile oils of AMB (A) and ACGD (B) by GC-MS. Classification and counts proportion of total 121 volatile organic compounds detected in AMB (C) and ACGD (D).

**Figure S3** The PC1 chart of total samples derived from PCA model (A) and correlation analysis of AMB and ACGD samples (B)

**Figure S4** The score plot (A), permutation plot (B), S-plot (C) derived from OPLS-DA model.

**Figure S5** The total ion chromatograms (TICs) of 16 tested internal standards

**Table S1** The detailed information of 121 volatile organic compounds in fresh bulbs of AMB and ACGD samples.

| **No.** | **Index** | **RT** | **Formula** | **Compounds** | **CAS** | **Match**  **factor** | **RI** | **Origin** |
| --- | --- | --- | --- | --- | --- | --- | --- | --- |
| Sulfur compounds | | | | | | | | |
| 1 | KMW0045 | 4.1894 | C2H6S2 | dimethyl disulfide | 624-92-0 | 87.9 | 730.9 | AMB/ACGD |
| 2 | XMW3428 | 7.683 | C6H8S | 3,4-dimethylthiophene | 632-15-5 | 96.0 | 901.9 | AMB/ACGD |
| 3 | NMW0777 | 7.9346 | C4H8S2 | 2-allyl methyl disulfide | 2179-58-0 | 93.0 | 914.9 | AMB/ACGD |
| 4 | GMW0062 | 8.7471 | C4H8S2 | methyl 1-propenyl disulfide | 5905-47-5 | 63.0 | 956.9 | AMB/ACGD |
| 5 | XMW3292 | 9.0374 | C5H5NOS | 2-thienylamide | 5813-89-8 | 57.2 | 971.9 | AMB/ACGD |
| 6 | GMW0113 | 11.4563 | C6H10S2 | diallyl disulfide | 2179-57-9 | 84.4 | 1100.9 | AMB/ACGD |
| 7 | GMW0101 | 11.7542 | C6H12S2 | propenyl propyl disulfide | 23838-20-2 | 92.6 | 1116.9 | AMB/ACGD |
| 8 | XMW2185 | 12.0146 | C5H10O2S | 2-methylthiolane 1,1-dioxide | 1003-46-9 | 61.8 | 1130.8 | AMB/ACGD |
| 9 | D384 | 12.216 | C4H8S3 | allyl methyl trisulfide | 34135-85-8 | 97.8 | 1141.6 | AMB/ACGD |
| 10 | GMW0115 | 12.4848 | C4H10S3 | methyl propyl trisulfide | 17619-36-2 | 95.2 | 1156.0 | AMB/ACGD |
| 11 | XMW0921 | 12.8313 | C9H12OS | 1-thien-2-ylpentan-1-one | 53119-25-8 | 73.3 | 1174.5 | AMB/ACGD |
| 12 | KMW0401 | 13.743 | C2H6S4 | dimethyl tetrasulfane | 5756-24-1 | 93.2 | 1223.4 | AMB/ACGD |
| 13 | XMW1414 | 14.3023 | C5H10N4S | 5-amino-2-ethyl-2,4-dihydro-4-methyl-3*H*-1,2,4-triazole-3-thione | 1000404-36-1 | 66.6 | 1253.5 | AMB/ACGD |
| 14 | XMW1061 | 14.5557 | C10H16S | 2-hexylthiophene | 18794-77-9 | 68.4 | 1267.2 | AMB/ACGD |
| 15 | KMW0352 | 14.7589 | C6H8OS2 | methyl 2-methyl-3-furyl disulfide | 65505-17-1 | 66.3 | 1278.1 | AMB/ACGD |
| 16 | D33 | 14.8082 | C8H10OS | 1-(2,5-dimethyl-3-thienyl)-ethanone | 2530-10-1 | 65.8 | 1280.7 | AMB/ACGD |
| 17 | GMW0108 | 15.2396 | C6H10S3 | diallyl trisulfide | 2050-87-5 | 90.1 | 1304.0 | AMB/ACGD |
| 18 | XMW0497 | 15.4054 | C6H4O2S | 2,5-thiophenedicarboxaldehyde | 932-95-6 | 59.4 | 1313.1 | AMB/ACGD |
| 19 | XMW3737 | 15.6004 | C8H8O2S | 5-acetyl-2-methyl-3-thiophenecarbaldehyde | 90345-55-4 | 79.1 | 1323.8 | AMB/ACGD |
| 20 | WMW0071 | 15.7513 | C6H14S3 | dipropyl trisulfane | 6028-61-1 | 75.9 | 1332.1 | AMB/ACGD |
| 21 | GMW0100 | 16.1063 | C6H12S3 | (*E*)-propenyl propyl trisulfide | 23838-27-9 | 89.7 | 1351.5 | AMB/ACGD |
| 22 | XMW2525 | 16.1824 | C11H12S | 2-propylbenzo[b]thiophene | 16587-32-9 | 47.4 | 1355.7 | AMB/ACGD |
| 23 | XMW2221 | 17.3614 | C7H9NS | 4-(methyl-mercapto)-aniline | 104-96-1 | 43.4 | 1421.0 | AMB/ACGD |
| 24 | XMW0533 | 17.4974 | C9H8S | 3-methyl-1-thiaindene | 1455-18-1 | 74.5 | 1428.8 | AMB/ACGD |
| 25 | XMW3650 | 17.9644 | H4N2O2S | sulfamide | 7803-58-9 | 50.7 | 1455.4 | AMB/ACGD |
| 26 | D268 | 19.8179 | C6H10S4 | diallyl tetrasulfide | 2444-49-7 | 65.5 | 1563.5 | AMB/ACGD |
| 27 | KMW0600 | 21.9049 | C10H10O2S2 | bis-(2-methyl-3-furyl) disulfide | 28588-75-2 | 49.0 | 1705.4 | AMB/ACGD |
| 28 | XMW1702 | 23.9748 | C10H11NO3S2 | 3-ethoxy-2-(2-thienylmethylsulfonyl)-propennitrile | 1000267-97-5 | 54.6 | 1980.9 | AMB |
| Heterocyclic compound | | | | | | | | |
| 29 | XMW0435 | 3.6286 | C6H8O | 2,4-dimethylfuran | 3710-43-8 | 92.7 | 701.8 | AMB/ACGD |
| 30 | XMW2995 | 7.0071 | C2H4N4 | 5-methyl-1*H*-tetrazole | 4076-36-2 | 71.4 | 870.0 | AMB/ACGD |
| 31 | NMW0017 | 7.3903 | C5H9NO | *N*-methyl-pyrrolidone | 872-50-4 | 65.1 | 888.0 | AMB/ACGD |
| 32 | D211 | 7.6561 | C4H10N2 | piperazine | 110-85-0 | 69.8 | 900.5 | AMB/ACGD |
| 33 | KMW0182 | 9.3865 | C9H14O | 2-amylfuran | 3777-69-3 | 98.9 | 990.0 | AMB/ACGD |
| 34 | WMW0081 | 9.5472 | C9H12O | cis-2-(2-pentenyl)-furan | 70424-13-4 | 79.3 | 998.3 | AMB/ACGD |
| 35 | XMW2266 | 9.6315 | C5H8O2 | γ-valerolactone | 108-29-2 | 61.4 | 1002.8 | AMB/ACGD |
| 36 | XMW1685 | 11.1237 | C8H13NO2 | 3,4-methyl-propyl-succinimide | 1000248-78-0 | 65.6 | 1083.0 | AMB/ACGD |
| 37 | KMW0286 | 11.1993 | C8H12N2O | isopropyl methoxy pyrazine | 25773-40-4 | 95.3 | 1087.1 | AMB/ACGD |
| 38 | KMW0380 | 12.8534 | C9H14N2O | 2-isobutyl-3-methoxypyrazine | 24683-00-9 | 65.8 | 1175.7 | AMB/ACGD |
| 39 | XMW0546 | 13.1709 | C11H21N | (5*R*,8a*R*)-5-propyloctahydroindolizine | 120057-35-4 | 60.8 | 1192.7 | AMB/ACGD |
| 40 | XMW0740 | 13.214 | C7H7N3 | 3-methyl-pyrrolo(2,3-b)-pyrazine | 20321-99-7 | 55.3 | 1195.0 | AMB/ACGD |
| 41 | XMW0163 | 15.5458 | C10H16O | 2-hexylfuran | 3777-70-6 | 66.3 | 1320.8 | AMB/ACGD |
| 42 | XMW1134 | 16.6066 | C9H16N2 | 4-butyl-3,4-dihydro-5-methyl-pyrimidine | 1000115-50-2 | 68.1 | 1378.9 | AMB/ACGD |
| 43 | XMW2869 | 17.6239 | C4H8N2O2 | N'-acetyl-acetohydrazide | 3148-73-0 | 57.2 | 1436.0 | AMB/ACGD |
| 44 | XMW1877 | 18.1276 | C11H14N4 | 4-(1-methyl-propyl-amino)-pyrido-[3,2-c]-pyridazine | 1000326-90-2 | 68.9 | 1464.7 | AMB/ACGD |
| 45 | NMW0335 | 19.75 | C9H7NO | quinolone | 59-31-4 | 45.7 | 1559.5 | AMB |
| 46 | XMW2946 | 20.223 | C6H6N2O2 | 6-hydroxynicotinamide | 3670-59-5 | 52.6 | 1587.5 | AMB/ACGD |
| 47 | XMW1703 | 20.9401 | C13H15NO3 | (2,3-dihydro-5-benzofuryl)-(4-morpholyl)-methanone | 1000268-71-6 | 69.7 | 1635.7 | AMB/ACGD |
| 48 | XMW3286 | 21.1559 | C13H22O2 | 5-methyl-2-octylfuran-3-one | 57877-72-2 | 98.1 | 1650.9 | AMB/ACGD |
| 49 | XMW0205 | 21.3589 | C15H10N2 | benzimidazo[2,1-a]-isoquinoline | 239-44-1 | 59.9 | 1665.2 | AMB/ACGD |
| 50 | XMW0955 | 21.5297 | C8H8N2O | 4-formyl-3,5-dimethyl-1H-pyrrole-2-carbonitrile | 1000296-05-3 | 55.3 | 1677.2 | AMB/ACGD |
| 51 | NMW0424 | 21.7353 | C12H10O4 | 7-acetoxy-4-methylcoumarin | 2747-05-9 | 65.7 | 1691.7 | AMB |
| 52 | XMW2407 | 22.7953 | C12H16N2O | 1-amino-4,4-dimethyl-1-(3-pyridyl)-pent-1-en-3-one | 136346-90-2 | 57.2 | 1800.2 | AMB/ACGD |
| 53 | XMW2657 | 23.2904 | C3H3N3O2 | 4-nitro-1*H*-pyrazole | 2075-46-9 | 62.0 | 1869.6 | AMB/ACGD |
| 54 | XMW0055 | 23.5483 | C17H24O3 | 7,9-di-tert-butyl-1-oxaspiro-(4,5)-deca-6,9-diene-2,8-dione | 82304-66-3 | 53.0 | 1907.2 | AMB/ACGD |
| Ester | | | | | | | | |
| 55 | XMW0574 | 10.8116 | C8H14O2 | pent-4-en-1-ol-propanoic acid | 30563-30-5 | 72.8 | 1066.3 | AMB/ACGD |
| 56 | XMW1127 | 10.8757 | C9H18O2 | formic acid octyl ester | 112-32-3 | 80.8 | 1069.7 | AMB/ACGD |
| 57 | XMW0969 | 11.3109 | C9H16O2 | 2-butenoic acid-2-methyl-2-methylpropyl ester | 66917-61-1 | 68.7 | 1093.1 | AMB/ACGD |
| 58 | XMW1085 | 12.7189 | C8H14O2 | butyl 2-butenoate | 7299-91-4 | 50.1 | 1168.5 | AMB/ACGD |
| 59 | XMW3765 | 12.9593 | C10H18O2 | vinyl 2-ethylhexanoate | 94-04-2 | 69.3 | 1181.3 | AMB/ACGD |
| 60 | XMW3158 | 13.3021 | C9H16O4 | 1-propanoyloxypropyl propanoate | 5331-24-8 | 57.4 | 1199.7 | AMB/ACGD |
| 61 | XMW1848 | 13.8407 | C8H13NO4 | d-proline-*N*-methoxycarbonyl-methyl ester | 1000320-78-5 | 63.8 | 1228.7 | AMB/ACGD |
| 62 | XMW2714 | 16.5238 | C10H12O2 | propyl benzoate | 2315-68-6 | 63.1 | 1374.4 | AMB/ACGD |
| 63 | XMW0506 | 18.4246 | C10H11NO4 | D-alanine-*N*-propargyl-oxycarbonyl-propargyl ester | 1000347-74-5 | 68.2 | 1481.6 | AMB/ACGD |
| 64 | XMW1046 | 18.9471 | C14H26O3 | heptanoic acid anhydride | 626-27-7 | 70.0 | 1511.8 | AMB/ACGD |
| 65 | XMW2167 | 19.7648 | C15H22O3 | 6-methoxythymyl isobutyrate | 1000413-80-3 | 71.3 | 1560.3 | AMB/ACGD |
| 66 | XMW0473 | 20.095 | C13H18O2 | hexyl benzoate | 6789-88-4 | 89.1 | 1579.9 | AMB/ACGD |
| 67 | XMW1259 | 21.6545 | C14H20O2 | benzoic acid hept-2-yl ester | 1000368-69-4 | 71.7 | 1686.0 | AMB/ACGD |
| 68 | KMW0689 | 22.5563 | C14H12O2 | benzyl benzoate | 120-51-4 | 81.4 | 1774.7 | AMB/ACGD |
| 69 | XMW2038 | 23.2558 | C21H32O4 | diethyl malonic acid hexyl phenethyl ester | 1000369-55-0 | 79.6 | 1864.8 | AMB/ACGD |
| 70 | XMW0017 | 23.6442 | C17H34O2 | methyl palmitate | 112-39-0 | 84.3 | 1923.7 | AMB/ACGD |
| 71 | XMW1827 | 23.8359 | C18H22O4 | phthalic acid-butyl hex-2-yn-4-yl ester | 1000315-19-2 | 81.4 | 1956.9 | AMB/ACGD |
| 72 | KMW0716 | 24.1885 | C19H38O2 | isopropyl palmitate | 142-91-6 | 71.2 | 2021.1 | AMB/ACGD |
| 73 | XMW2291 | 24.9484 | C20H40O2 | butyl palmitate | 111-06-8 | 67.6 | 2185.0 | AMB/ACGD |
| Aldehyde | | | | | | | | |
| 74 | KMW0066 | 5.4469 | C6H12O | hexanal | 66-25-1 | 97.6 | 796.3 | AMB/ACGD |
| 75 | XMW1375 | 6.1252 | C6H10O | 2-methyl-2-pentenal | 623-36-9 | 97.3 | 828.5 | AMB/ACGD |
| 76 | KMW0095 | 6.6434 | C6H10O | 2-hexenal | 505-57-7 | 85.7 | 852.9 | AMB/ACGD |
| 77 | GMW0099 | 8.772 | C7H12O | (*E*)-2-heptenal | 2463-63-0 | 84.2 | 958.2 | AMB/ACGD |
| 78 | XMW0483 | 8.5641 | C5H5NO | 2-formyl-1H-pyrrole | 1003-29-8 | 63.8 | 947.5 | AMB |
| 79 | KMW0253 | 10.6456 | C8H14O | 2-octenal | 2363-89-5 | 88.2 | 1057.3 | AMB/ACGD |
| 80 | WMW0047 | 10.1507 | C8H12O | 5-ethylcyclopentene-1-carbaldehyde | 36431-60-4 | 81.8 | 1030.7 | AMB/ACGD |
| 81 | KMW0212 | 10.4093 | C8H8O | phenylacetaldehyde | 122-78-1 | 75.1 | 1044.6 | AMB/ACGD |
| 82 | KMW0282 | 11.5132 | C9H18O | nonanal | 124-19-6 | 77.5 | 1104.0 | AMB/ACGD |
| 83 | KMW0399 | 13.4094 | C10H20O | decanal | 112-31-2 | 95.7 | 1205.5 | AMB/ACGD |
| 84 | KMW0407 | 13.6114 | C9H14O | (2*E*,4*E*)-2,4-nonadienal | 5910-87-2 | 84.0 | 1216.3 | AMB/ACGD |
| 85 | KMW0519 | 16.9348 | C8H8O3 | vanillin | 121-33-5 | 81.9 | 1396.9 | AMB/ACGD |
| 86 | XMW1714 | 22.2219 | C16H14O2 | 4-(1-phenyl-2-propenyloxy)-benzaldehyde | 1000277-56-1 | 57.8 | 1739.1 | AMB/ACGD |
| Phenol | | | | | | | | |
| 87 | NMW0105 | 10.5314 | C9H12O | 4-propylphenol | 645-56-7 | 70.8 | 1051.2 | AMB/ACGD |
| 88 | XMW3151 | 10.7936 | C9H12O | 3,4,5-trimethylphenol | 527-54-8 | 73.2 | 1065.3 | AMB |
| 89 | NMW0128 | 15.5167 | C10H14O | 4-(2-butyl)-phenol | 99-71-8 | 73.6 | 1319.2 | AMB/ACGD |
| 90 | NMW0153 | 12.1213 | C10H14O | 4-butylphenol | 1638-22-8 | 73.7 | 1136.5 | AMB/ACGD |
| 91 | XMW1108 | 18.7953 | C15H24O | 2,4,6-tri(propan-2-yl)-phenol | 2934-07-8 | 61.2 | 1502.8 | AMB/ACGD |
| 92 | XMW0507 | 19.0455 | C11H16O2 | olivetol | 500-66-3 | 52.1 | 1517.7 | AMB/ACGD |
| Alcohol | | | | | | | | |
| 93 | KMW0173 | 9.2065 | C8H16O | 1-octen-3-ol | 3391-86-4 | 93.8 | 980.7 | AMB/ACGD |
| 94 | KMW0523 | 12.0168 | C11H24O | 1-undecanol | 112-42-5 | 83.1 | 1130.9 | AMB/ACGD |
| 95 | XMW2288 | 14.4514 | C9H20O | (6*S*)-6-methyl-1-octanol | 110453-78-6 | 72.8 | 1261.5 | AMB/ACGD |
| 96 | KMW0376 | 15.1959 | C9H20O | 2-nonanol | 628-99-9 | 70.3 | 1301.6 | AMB/ACGD |
| 97 | XMW1077 | 16.4259 | C7H14O4 | 2,2'-(1,3-dioxolane-2,2-diyl)-diethanol | 5694-95-1 | 62.1 | 1369.0 | AMB |
| 98 | XMW0728 | 19.5117 | C12H16O | (1*R*,2*R*)-2-methyl-1-(4-methylphenyl)-3-buten-1-ol | 83173-76-6 | 49.1 | 1545.3 | AMB/ACGD |
| 99 | KMW0674 | 22.0021 | C12H24O | (*E*)-2-dodecen-1-ol | 69064-37-5 | 82.7 | 1715.8 | AMB/ACGD |
| 100 | NMW0339 | 24.3546 | C10H11NO | tryptophol | 526-55-6 | 44.8 | 2054.9 | AMB/ACGD |
| Ketone | | | | | | | | |
| 101 | XMW0637 | 4.8133 | C7H12O | 2,2,3-trimethylcyclobutanone | 1449-49-6 | 87.6 | 763.4 | AMB/ACGD |
| 102 | KMW0474 | 15.0034 | C11H22O | 2-undecanone | 112-12-9 | 97.6 | 1291.3 | AMB/ACGD |
| 103 | XMW0737 | 15.3914 | C9H10O2 | o-acetyl-p-cresol | 1450-72-2 | 87.7 | 1312.3 | AMB/ACGD |
| 104 | D380 | 17.6974 | C11H18O2 | 2-hexyl-5-methylfuran-3-one | 33922-66-6 | 99.4 | 1440.2 | AMB/ACGD |
| 105 | XMW0973 | 17.8051 | C9H13NO | methyl(2,4,5-trimethyl-1H-pyrrol-3-yl) ketone | 19005-95-9 | 57.2 | 1446.3 | AMB/ACGD |
| 106 | XMW0508 | 18.3836 | C10H12O3 | 2,5-dihydroxy-6-propan-2-ylcyclohepta-2,4,6-trien-1-one | 54755-56-5 | 61.2 | 1479.3 | AMB/ACGD |
| 107 | XMW1348 | 18.624 | C13H26O | 2-tridecanone | 593-08-8 | 81.8 | 1492.9 | AMB/ACGD |
| Aromatics | | | | | | | | |
| 108 | XMW0581 | 12.2782 | C8H10O2 | 1,2-dimethoxybenzene | 91-16-7 | 83.7 | 1144.9 | AMB/ACGD |
| 109 | XMW0287 | 15.7379 | C11H14O2 | isobutyl benzoate | 120-50-3 | 64.9 | 1331.3 | AMB/ACGD |
| 110 | NMW0266 | 22.0959 | C12H16O3 | β-asarone | 5273-86-9 | 49.6 | 1725.7 | AMB/ACGD |
| 111 | NMW0284 | 23.4015 | C13H10O | xanthene | 92-83-1 | 54.2 | 1885.2 | AMB/ACGD |
| Hydrocarbons | | | | | | | | |
| 112 | XMW2460 | 9.7962 | C8H14 | 1-ethyl-cyclohexene | 1453-24-3 | 82.3 | 1011.6 | AMB/ACGD |
| 113 | KMW0727 | 24.5737 | C21H44 | heneicosane | 629-94-7 | 96.4 | 2099.5 | AMB/ACGD |
| 114 | ZMW0001 | 25.4392 | C23H48 | tricosane | 638-67-5 | 94.9 | 2298.4 | AMB/ACGD |
| Acid | | | | | | | | |
| 115 | XMW0026 | 11.0564 | C7H14O2 | n-heptanoic acid | 111-14-8 | 81.2 | 1079.4 | AMB/ACGD |
| 116 | WMW0131 | 22.4119 | C14H28O2 | myristic acid | 544-63-8 | 72.0 | 1759.4 | AMB/ACGD |
| 117 | WMW0132 | 23.2078 | C15H30O2 | pentadecanoic acid | 1002-84-2 | 66.5 | 1858.0 | AMB/ACGD |
| Amine | | | | | | | | |
| 118 | XMW1409 | 9.877 | C9H13N | (*R*)-α,p-dimethylbenzylamine | 4187-38-6 | 63.2 | 1016.0 | AMB/ACGD |
| 119 | XMW0124 | 11.8655 | C8H7N | 3-aminophenylacetylene | 54060-30-9 | 52.7 | 1122.8 | AMB/ACGD |
| Terpenoids | | | | | | | | |
| 120 | KMW0370 | 14.1023 | C11H20O | methyl isoborneol | 2371-42-8 | 69.0 | 1242.8 | AMB/ACGD |
| 121 | XMW2159 | 14.8714 | C11H20O | 4-(2-methoxypropan-2-yl)-1-methylcyclohex-1-ene | 1000411-43-4 | 60.1 | 1284.1 | AMB/ACGD |

Note: RT, retention time; RI, retention index.

**Table S2** Content levels (μg/g) of VOCs in AMB and ACGD samples.

| **Index** | **Compounds** | **Content level (μg/g)** | | | | | |
| --- | --- | --- | --- | --- | --- | --- | --- |
|  |  | **AMB1** | **AMB2** | **AMB3** | **ACGD1** | **ACGD2** | **ACGD3** |
| KMW0045 | dimethyl disulfide | 27.1368 | 48.6611 | 17.2508 | 125.7838 | 111.6142 | 87.7992 |
| XMW3428 | 3,4-dimethylthiophene | 764.7914 | 700.6001 | 583.1196 | 73.2503 | 41.5718 | 48.3890 |
| NMW0777 | 2-allyl methyl disulfide | 4.8631 | 7.9383 | 3.9800 | 83.3362 | 36.3259 | 38.1769 |
| GMW0062 | methyl 1-propenyl disulfide | 2.5161 | 3.0170 | 2.1742 | 2.1357 | 2.2480 | 1.2526 |
| XMW3292 | 2-thienylamide | 51.7132 | 76.1740 | 37.2575 | 111.7585 | 186.0654 | 82.9321 |
| GMW0113 | diallyl disulfide | 18.9211 | 17.2404 | 15.2038 | 26.7171 | 11.9174 | 14.1758 |
| GMW0101 | propenyl propyl disulfide | 121.0223 | 97.6700 | 75.5991 | 39.2740 | 35.3562 | 32.2201 |
| XMW2185 | 2-methylthiolane 1,1-dioxide | 7.8598 | 5.1389 | 6.4635 | 0.3057 | 0.2530 | 0.2733 |
| D384 | allyl methyl trisulfide | 13.4604 | 23.7661 | 11.9986 | 176.1280 | 120.5826 | 83.8710 |
| GMW0115 | methyl propyl trisulfide | 230.2767 | 260.7021 | 160.1430 | 185.8131 | 176.5401 | 108.6422 |
| XMW0921 | 1-thien-2-ylpentan-1-one | 0.0498 | 0.0313 | 0.0393 | 1.6888 | 1.8325 | 1.8480 |
| KMW0401 | dimethyl tetrasulfane | 94.0890 | 175.5607 | 82.8904 | 196.7534 | 207.1247 | 153.6500 |
| XMW1414 | 5-amino-2-ethyl-2,4-dihydro-4-methyl-3*H*-1,2,4-triazole-3-thione | 1.6919 | 2.0547 | 1.3180 | 3.9690 | 2.7875 | 2.6735 |
| XMW1061 | 2-hexylthiophene | 20.6211 | 21.4708 | 20.1689 | 8.3351 | 9.4573 | 6.6261 |
| KMW0352 | methyl 2-methyl-3-furyl disulfide | 1.7767 | 2.1766 | 1.1225 | 0.3241 | 0.1729 | 0.2354 |
| D33 | 1-(2,5-dimethyl-3-thienyl)-ethanone | 5.3841 | 3.9987 | 4.4331 | 0.2525 | 0.1114 | 0.1569 |
| GMW0108 | diallyl trisulfide | 2.2057 | 2.0507 | 2.0971 | 6.1834 | 1.4937 | 1.7496 |
| XMW0497 | 2,5-thiophenedicarboxaldehyde | 6.0335 | 7.6339 | 4.2236 | 0.0955 | 0.0273 | 0.0412 |
| XMW3737 | 5-acetyl-2-methyl-3-thiophenecarbaldehyde | 2.3273 | 1.8016 | 1.9110 | 0.2228 | 0.2640 | 0.1883 |
| WMW0071 | dipropyl trisulfane | 40.4225 | 32.9195 | 27.7144 | 14.3763 | 9.2307 | 9.6422 |
| GMW0100 | (*E*)-propenyl propyl trisulfide | 97.1359 | 101.2061 | 94.8803 | 3.5129 | 1.5221 | 1.9509 |
| XMW2525 | 2-propylbenzo[b]thiophene | 0.5448 | 0.2714 | 0.2842 | 0.0880 | 0.0772 | 0.0651 |
| XMW2221 | 4-(methyl-mercapto)-aniline | 0.2768 | 0.1984 | 0.2315 | 0.3536 | 0.2799 | 0.5031 |
| XMW0533 | 3-methyl-1-thiaindene | 1.2445 | 1.7212 | 1.4968 | 1.6815 | 0.7547 | 0.1473 |
| XMW3650 | sulfamide | 17.4340 | 11.5240 | 13.9621 | 15.6177 | 16.4479 | 18.8190 |
| D268 | diallyl tetrasulfide | 1.4316 | 1.3229 | 1.1550 | 0.4173 | 0.1386 | 0.2531 |
| KMW0600 | bis(2-methyl-3-furyl)-disulfide | 1.0997 | 1.0089 | 0.9169 | 0.1388 | 0.0860 | 0.0760 |
| XMW1702 | 3-ethoxy-2-(2-thienylmethylsulfonyl)-propennitrile | 2.9783 | 2.8058 | 2.7055 | 0.0001 | 0.0001 | 0.0001 |
| XMW0435 | 2,4-dimethylfuran | 37.0175 | 36.5008 | 28.9997 | 0.9095 | 0.3438 | 0.3816 |
| XMW2995 | 5-methyl-1H-tetrazole | 0.9879 | 0.4369 | 0.8297 | 0.9356 | 0.9428 | 1.5100 |
| NMW0017 | *N*-methyl-pyrrolidone | 1.6683 | 1.5099 | 1.0127 | 0.2710 | 0.1389 | 0.1867 |
| D211 | piperazine | 1.8573 | 1.7857 | 1.6719 | 2.4954 | 2.2809 | 3.3079 |
| KMW0182 | 2-amylfuran | 43.7449 | 26.3151 | 44.3646 | 34.6497 | 46.2071 | 32.0886 |
| WMW0081 | *cis*-2-(2-pentenyl)-furan | 0.4772 | 0.2943 | 0.3810 | 0.2673 | 0.2926 | 0.2048 |
| XMW2266 | γ-valerolactone | 1.1846 | 0.7850 | 1.0279 | 0.9067 | 0.8034 | 0.8509 |
| XMW1685 | 3,4-methylpropylsuccinimide | 8.4892 | 9.0262 | 7.3643 | 12.7655 | 10.7101 | 12.5254 |
| KMW0286 | isopropyl methoxy pyrazine | 13.8998 | 10.0530 | 13.9501 | 3.6574 | 1.6767 | 2.9878 |
| KMW0380 | 2-isobutyl-3-methoxypyrazine | 0.0498 | 0.0313 | 0.0393 | 1.6888 | 1.8325 | 1.8480 |
| XMW0546 | (5*R*,8a*R*)-5-propyloctahydroindolizine | 39.5223 | 35.8097 | 39.4159 | 48.2823 | 49.0344 | 50.3963 |
| XMW0740 | 3-methyl-pyrrolo(2,3-b)-pyrazine | 4.6184 | 5.8090 | 4.3760 | 0.5403 | 0.4040 | 0.3784 |
| XMW0163 | 2-hexylfuran | 3.6647 | 4.7541 | 3.2684 | 22.0498 | 25.8545 | 17.7638 |
| XMW1134 | 4-butyl-3,4-dihydro-5-methyl-pyrimidine | 0.3265 | 0.2144 | 0.2796 | 3.0361 | 2.4206 | 5.2971 |
| XMW2869 | *N'*-acetyl-acetohydrazide | 36.9545 | 46.4701 | 23.7128 | 7.1920 | 10.4150 | 3.3050 |
| XMW1877 | 4-(1-methylpropyl-amino)-pyrido-[3,2-c]-pyridazine | 0.7901 | 0.6682 | 0.7760 | 0.0975 | 0.0161 | 0.0515 |
| NMW0335 | quinolone | 0.7170 | 0.9142 | 0.7614 | 0.0001 | 0.0001 | 0.0001 |
| XMW2946 | 6-hydroxynicotinamide | 2.9496 | 3.6780 | 2.7985 | 0.9311 | 0.9107 | 1.3653 |
| XMW1703 | (2,3-dihydro-5-benzofuryl)-(4-morpholyl)-methanone | 8.1738 | 10.2986 | 7.7541 | 1.1742 | 0.6425 | 1.2323 |
| XMW3286 | 5-methyl-2-octylfuran-3-one | 2.7146 | 1.2963 | 1.2714 | 147.4282 | 59.3983 | 231.8608 |
| XMW0205 | benzimidazo[2,1-a]-isoquinoline | 8.3688 | 7.2744 | 6.1079 | 1.6839 | 1.0856 | 0.9374 |
| XMW0955 | 4-formyl-3,5-dimethyl-1H-pyrrole-2-carbonitrile | 0.4951 | 0.7186 | 0.4631 | 0.1640 | 0.0670 | 0.0991 |
| NMW0424 | 7-acetoxy-4-methylcoumarin | 0.9591 | 1.0038 | 0.8800 | 0.0001 | 0.0001 | 0.0001 |
| XMW2407 | 1-amino-4,4-dimethyl-1-(3-pyridyl)-pent-1-en-3-one | 0.1834 | 0.1215 | 0.1478 | 0.1128 | 0.1202 | 0.0413 |
| XMW2657 | 4-nitro-1H-pyrazole | 1.0352 | 0.3956 | 0.3958 | 0.0872 | 0.0580 | 0.0844 |
| XMW0055 | 7,9-di-tert-butyl-1-oxaspiro-(4,5)deca-6,9-diene-2,8-dione | 0.1190 | 0.1347 | 0.1201 | 0.0867 | 0.0373 | 0.0500 |
| XMW0574 | pent-4-en-1-ol-propanoic acid | 0.7159 | 0.6985 | 0.6639 | 0.3897 | 0.5123 | 0.4079 |
| XMW1127 | formic acid octyl ester | 0.5504 | 0.4797 | 0.5880 | 0.3917 | 0.6431 | 0.5507 |
| XMW0969 | 2-butenoic acid-2-methyl-2-methylpropyl ester | 10.5986 | 7.2716 | 7.3076 | 1.9023 | 2.4259 | 2.7167 |
| XMW1085 | butyl 2-butenoate | 10.5622 | 9.2262 | 12.4162 | 14.8043 | 13.4637 | 16.6681 |
| XMW3765 | vinyl 2-ethylhexanoate | 0.5255 | 0.7110 | 0.3709 | 4.2052 | 2.0437 | 4.1218 |
| XMW3158 | 1-propanoyloxypropyl propanoate | 0.7814 | 0.4208 | 0.5473 | 1.8881 | 1.4893 | 0.9670 |
| XMW1848 | d-proline-*N*-methoxycarbonyl-methyl ester | 24.4031 | 23.2782 | 18.0590 | 1.3418 | 0.6956 | 0.9407 |
| XMW2714 | propyl benzoate | 0.2366 | 0.1943 | 0.1940 | 0.1313 | 0.1426 | 0.1343 |
| XMW0506 | D-alanine-*N*-propargyloxy-carbonyl-propargyl ester | 2.7905 | 3.5152 | 4.8591 | 10.6127 | 8.8143 | 7.6117 |
| XMW1046 | heptanoic acid anhydride | 1.7905 | 2.6069 | 1.1165 | 1.8253 | 0.8010 | 1.5519 |
| XMW2167 | 6-methoxythymyl isobutyrate | 0.3518 | 0.6591 | 0.2906 | 0.3981 | 0.2370 | 0.2151 |
| XMW0473 | hexyl benzoate | 0.0727 | 0.0456 | 0.0485 | 24.2893 | 8.6376 | 43.7865 |
| XMW1259 | benzoic acid hept-2-yl ester | 0.3037 | 0.3708 | 0.2694 | 3.3837 | 2.2433 | 5.9792 |
| KMW0689 | benzyl benzoate | 0.2050 | 0.2770 | 0.2269 | 1.7528 | 2.1123 | 6.5630 |
| XMW2038 | diethyl-malonic acid hexyl phenethyl ester | 0.3725 | 0.3388 | 0.3276 | 2.4615 | 0.7463 | 5.7751 |
| XMW0017 | methyl palmitate | 1.6823 | 1.3154 | 1.8743 | 1.6067 | 1.7170 | 1.2333 |
| XMW1827 | phthalic acid-butyl hex-2-yn-4-yl ester | 9.2473 | 7.5924 | 8.9207 | 1.8250 | 1.7727 | 1.6076 |
| KMW0716 | isopropyl palmitate | 0.3578 | 0.3540 | 0.3349 | 0.1305 | 0.1458 | 0.1154 |
| XMW2291 | butyl palmitate | 0.1259 | 0.1477 | 0.1438 | 0.0729 | 0.1023 | 0.0447 |
| KMW0066 | hexanal | 46.4111 | 33.1376 | 48.9723 | 62.2755 | 67.0233 | 59.2184 |
| XMW1375 | 2-methyl-2-pentenal | 510.7507 | 389.2327 | 424.9828 | 12.5432 | 7.9506 | 9.7542 |
| KMW0095 | 2-hexenal | 0.3326 | 0.2369 | 0.3104 | 0.6139 | 0.5828 | 0.6135 |
| GMW0099 | (*E*)-2-heptenal | 2.3587 | 2.2943 | 2.1720 | 3.4331 | 3.9022 | 3.0417 |
| XMW0483 | 2-formyl-1H-pyrrole | 1.8781 | 1.5980 | 1.2931 | 0.0001 | 0.0001 | 0.0001 |
| KMW0253 | 2-octenal | 2.3490 | 1.6468 | 2.3243 | 4.8553 | 5.7630 | 4.3904 |
| WMW0047 | 5-ethylcyclopentene-1-carbaldehyde | 0.7131 | 0.5768 | 0.7073 | 1.8977 | 2.3128 | 1.5076 |
| KMW0212 | phenylacetaldehyde | 2.1876 | 1.5568 | 1.6534 | 4.0711 | 4.7366 | 3.3497 |
| KMW0282 | nonanal | 13.5216 | 10.1905 | 12.3350 | 4.7839 | 5.9284 | 5.3713 |
| KMW0399 | decanal | 4.3474 | 3.8448 | 4.3018 | 1.3347 | 1.6095 | 1.4166 |
| KMW0407 | (2*E*,4*E*)-2,4-nonadienal | 0.8462 | 0.7058 | 1.0033 | 4.2076 | 5.7046 | 3.7797 |
| KMW0519 | vanillin | 9.2745 | 11.0863 | 7.9064 | 4.5816 | 3.1047 | 1.8042 |
| XMW1714 | 4-(1-phenyl-2-propenyloxy)-benzaldehyde | 2.0262 | 1.6339 | 1.5937 | 0.2055 | 0.1107 | 0.1437 |
| NMW0105 | 4-propylphenol | 2.0923 | 2.6997 | 1.9801 | 1.2063 | 0.8713 | 0.8582 |
| XMW3151 | 3,4,5-trimethylphenol | 0.8375 | 0.9845 | 0.6984 | 0.0001 | 0.0001 | 0.0001 |
| NMW0128 | 4-(2-butyl)-phenol | 9.6989 | 12.2295 | 6.9408 | 7.0080 | 2.7612 | 3.7207 |
| NMW0153 | 4-butylphenol | 2.0816 | 2.4621 | 2.7364 | 1.5232 | 1.5417 | 0.9955 |
| XMW1108 | 2,4,6-tri(propan-2-yl)-phenol | 1.8277 | 1.4275 | 1.6449 | 0.1550 | 0.0845 | 0.2035 |
| XMW0507 | olivetol | 0.2696 | 0.2223 | 0.2461 | 1.9671 | 1.8205 | 1.5635 |
| KMW0173 | 1-octen-3-ol | 2.7243 | 2.2272 | 2.6056 | 1.4199 | 2.1091 | 1.2943 |
| KMW0523 | 1-undecanol | 8.3590 | 5.4423 | 6.8641 | 0.4144 | 0.2995 | 0.3416 |
| XMW2288 | (6*S*)-6-methyl-1-octanol | 0.2985 | 0.2460 | 0.2788 | 1.2541 | 1.9786 | 1.1543 |
| KMW0376 | 2-nonanol | 1.7860 | 2.1683 | 1.5347 | 2.4100 | 1.7025 | 4.5433 |
| XMW1077 | 2,2'-(1,3-dioxolane-2,2-diyl)-diethanol | 0.5771 | 1.1199 | 0.7609 | 0.0001 | 0.0001 | 0.0001 |
| XMW0728 | (1*R*,2*R*)-2-methyl-1-(4-methylphenyl)-3-buten-1-ol | 1.5404 | 1.5981 | 1.1317 | 2.1574 | 0.5308 | 0.6588 |
| KMW0674 | (*E*)-2-dodecen-1-ol | 1.2061 | 1.0083 | 1.0436 | 0.2760 | 0.1841 | 0.2210 |
| NMW0339 | tryptophol | 0.2001 | 0.2324 | 0.2211 | 0.0072 | 0.0042 | 0.0058 |
| XMW0637 | 2,2,3-trimethylcyclobutanone | 0.6495 | 0.9305 | 0.6325 | 1.6607 | 1.7820 | 1.1875 |
| KMW0474 | 2-undecanone | 0.6609 | 0.4914 | 0.3585 | 14.1674 | 8.8984 | 42.7440 |
| XMW0737 | o-acetyl-p-cresol | 13.4692 | 12.3562 | 12.3333 | 7.7681 | 6.9037 | 5.5238 |
| D380 | 2-hexyl-5-methylfuran-3-one | 4.7970 | 7.9237 | 5.2381 | 389.4758 | 240.0833 | 833.6891 |
| XMW0973 | methyl-(2,4,5-trimethyl-1*H*-pyrrol-3-yl) ketone | 1.8195 | 1.2816 | 1.7760 | 2.6325 | 2.2168 | 2.1057 |
| XMW0508 | 2,5-dihydroxy-6-propan-2-ylcyclohepta-2,4,6-trien-1-one | 127.9448 | 112.5892 | 128.8369 | 167.8635 | 156.9171 | 185.0881 |
| XMW1348 | 2-tridecanone | 0.4175 | 0.2854 | 0.4731 | 7.6956 | 4.8514 | 17.7372 |
| XMW0581 | 1,2-dimethoxybenzene | 7.8593 | 8.2678 | 6.9037 | 0.1686 | 0.1097 | 0.1186 |
| XMW0287 | isobutyl benzoate | 3.6015 | 3.5205 | 2.8021 | 7.4626 | 2.6263 | 4.9235 |
| NMW0266 | β-asarone | 0.1934 | 0.2606 | 0.2091 | 0.1734 | 0.0573 | 0.1741 |
| NMW0284 | xanthene | 1.1314 | 0.4790 | 0.6395 | 0.1038 | 0.0553 | 0.0532 |
| XMW2460 | 1-ethyl-cyclohexene | 2.2853 | 2.2190 | 2.0436 | 1.9274 | 1.8787 | 1.5954 |
| KMW0727 | heneicosane | 5.3090 | 5.4247 | 4.3429 | 0.4782 | 0.3309 | 0.1762 |
| ZMW0001 | tricosane | 10.1975 | 12.1130 | 9.7364 | 1.9494 | 2.1904 | 1.0433 |
| XMW0026 | n-heptanoic acid | 0.6476 | 0.9262 | 0.5510 | 6.3821 | 7.0730 | 26.7820 |
| WMW0131 | myristic acid | 1.0787 | 1.0453 | 1.0357 | 0.8449 | 0.6531 | 0.3020 |
| WMW0132 | pentadecanoic acid | 6.2312 | 5.7185 | 6.0887 | 0.9791 | 0.7751 | 0.4001 |
| XMW1409 | (*R*)-α,p-dimethylbenzylamine | 1.4085 | 0.7679 | 0.9147 | 0.0839 | 0.0416 | 0.0500 |
| XMW0124 | 3-aminophenylacetylene | 18.3404 | 15.9483 | 14.9947 | 0.9667 | 0.5223 | 0.5839 |
| KMW0370 | methyl isoborneol | 71.1067 | 69.2324 | 74.1638 | 76.3512 | 75.8195 | 77.4864 |
| XMW2159 | 4-(2-methoxypropan-2-yl)-1-methylcyclohex-1-ene | 1.2466 | 0.8432 | 1.1592 | 0.3379 | 0.1294 | 0.2259 |

**Table S3** The detailed information of 16 internal standards in AMB and ACGD samples.

| **IS** | **CAS** | **RT** | **RI** | **MF** | **AMB1** | **AMB2** | **AMB3** | **CV**  **(%)** | **ACGD1** | **ACGD2** | **ACGD3** | **CV**  **(%)** | **QC1** | **QC2** | **QC3** | **QC4** | **CV**  **(%)** |
| --- | --- | --- | --- | --- | --- | --- | --- | --- | --- | --- | --- | --- | --- | --- | --- | --- | --- |
| 3-Hexanone  -2,2,4,4-d4 | 24588-54-3 | 5.0 | 777.0 | 90.6 | 360286 | 144547 | 383771 | 45 | 302336 | 381821 | 380290 | 13 | 219257 | 338548 | 229532 | 87354 | 47 |
| 2-Picoline-d7 | 93951-93-0 | 5.8 | 814.0 | 78.5 | 1339066 | 1138539 | 1270289 | 8 | 1135758 | 1281690 | 1463389 | 13 | 626391 | 622629 | 485550 | 498980 | 14 |
| p-Xylene-d10 | 41051-88-1 | 6.9 | 863.8 | 86.2 | 1976028 | 555173 | 2178833 | 56 | 1503507 | 1572955 | 2273682 | 24 | 1161218 | 1641322 | 1366275 | 346242 | 49 |
| [3,4,5-2H3]-Furfural | 53599-40-9 | 7.4 | 888.3 | 86.6 | 43381 | 33593 | 19790 | 37 | 11039 | 6926 | 11582 | 26 | 5992 | 19357 | 4794 | 11463 | 64 |
| Benzaldehyde-d6 | 17901-93-8 | 8.9 | 962.4 | 93.0 | 1034196 | 897217 | 936000 | 7 | 1204836 | 1620797 | 1523524 | 15 | 825078 | 835198 | 660684 | 586134 | 17 |
| **[2H8]-Acetophenone** | **19547-00-3** | **10.7** | **1060.9** | **96.0** | **2853228** | **2392029** | **2938047** | **11** | **3455888** | **4384985** | **4167108** | **12** | **2189763** | **2251635** | **1709189** | **1560563** | **18** |
| [3,4,5,6-2H4,OD]-2  -Methoxyphenol | 20189-11-1 | 11.1 | 1083.2 | 75.3 | 306303 | 499835 | 355213 | 26 | 104936 | 115005 | 88589 | 13 | 412303 | 413252 | 311136 | 366927 | 13 |
| (+/-)-linalool-d3 | 1216673-02-7 | 11.4 | 1098.3 | 88.5 | 1656549 | 1195221 | 1690882 | 18 | 2050522 | 2074363 | 2156689 | 3 | 1846790 | 2207801 | 2008330 | 1588187 | 14 |
| [2H4]-n-Nonyl-Alcohol | 33975-46-1 | 12.7 | 1168.9 | 80.4 | 1174527 | 835462 | 1215842 | 19 | 1268495 | 1541740 | 2070742 | 25 | 3776328 | 4589954 | 3948581 | 3408664 | 13 |
| [3,4,5,6-2H4]-Methyl  -2-Hydroxybenzoate | 1219802-12-6 | 13.2 | 1193.0 | 97.4 | 2255928 | 1708902 | 2316888 | 16 | 3338491 | 4293276 | 4205748 | 13 | 3626573 | 3936090 | 3008487 | 2421163 | 21 |
| (R)-(-)-Carvone  -4,4,6,6-d4 | IR-37376 | 14.1 | 1242.9 | 98.8 | 4049302 | 3267103 | 4307725 | 14 | 5265951 | 6649705 | 6446174 | 12 | 3033371 | 3161568 | 2505650 | 2166212 | 17 |
| 4-Hydroxy-3-methoxy  -d3 benzaldehyde | 74495-74-2 | 16.9 | 1394.9 | 78.8 | 123849 | 155701 | 284890 | 45 | 374389 | 149625 | 183293 | 51 | 129031 | 149211 | 153526 | 175756 | 13 |
| [2H25]-n-Dodecanol | 160776-83-0 | 18.0 | 1455.2 | 92.0 | 997357 | 553832 | 819214 | 28 | 1075621 | 1448336 | 1574650 | 19 | 706273 | 758649 | 555000 | 485630 | 20 |
| [2H3]-beta-Ionone | 217482-81-0 | 18.4 | 1479.0 | 91.8 | 1797226 | 1293273 | 1927896 | 20 | 2995370 | 3508869 | 3803270 | 12 | 2088149 | 2080265 | 1668172 | 1523872 | 16 |
| Methyl Jasmonate  -d5 | D-6710 | 21.1 | 1643.3 | 76.9 | 237923 | 157836 | 238097 | 22 | 340971 | 456226 | 465371 | 16 | 203883 | 197503 | 135236 | 141939 | 21 |
| [2H31]-Ethyl  -Hexadecanoate | 1215721-57-5 | 23.9 | 1966.2 | 77.8 | 1172405 | 1007287 | 1304061 | 13 | 1197065 | 884478 | 1099467 | 15 | 349791 | 489412 | 476990 | 454965 | 14 |

Note: RT, retention time; RI, retention index; MF, match factor; CV, coefficient of variation.


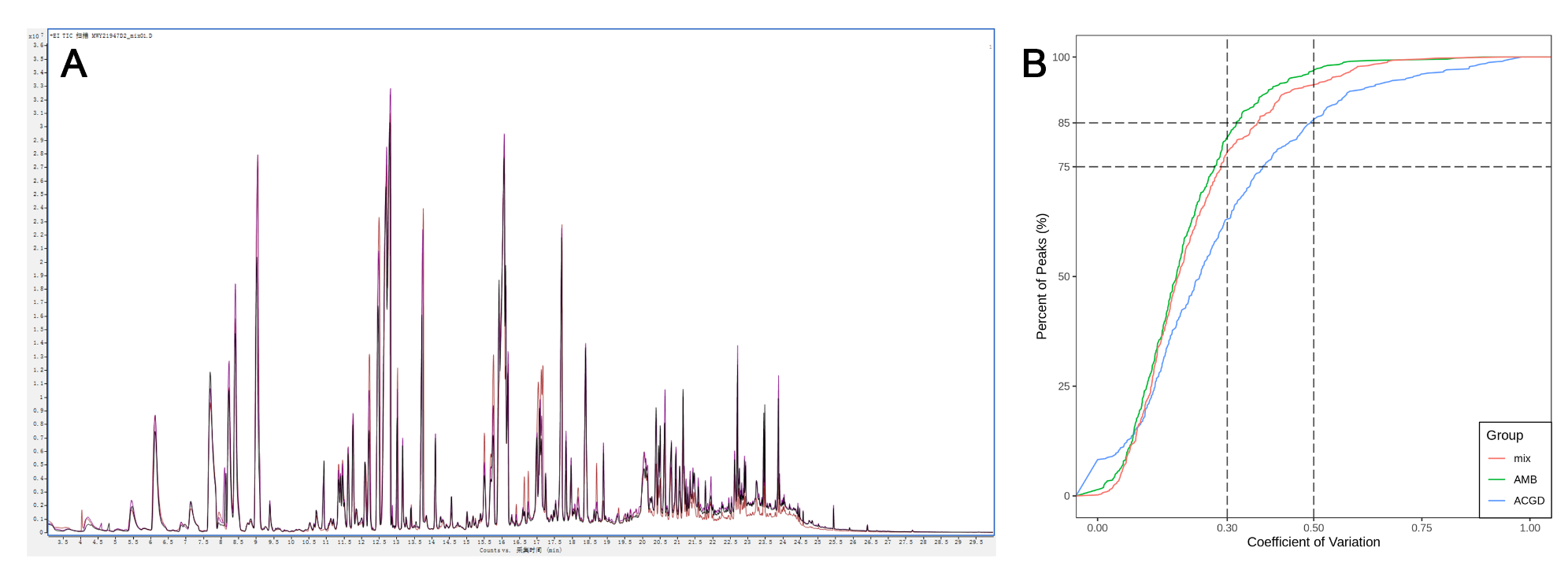


**Figure S1**


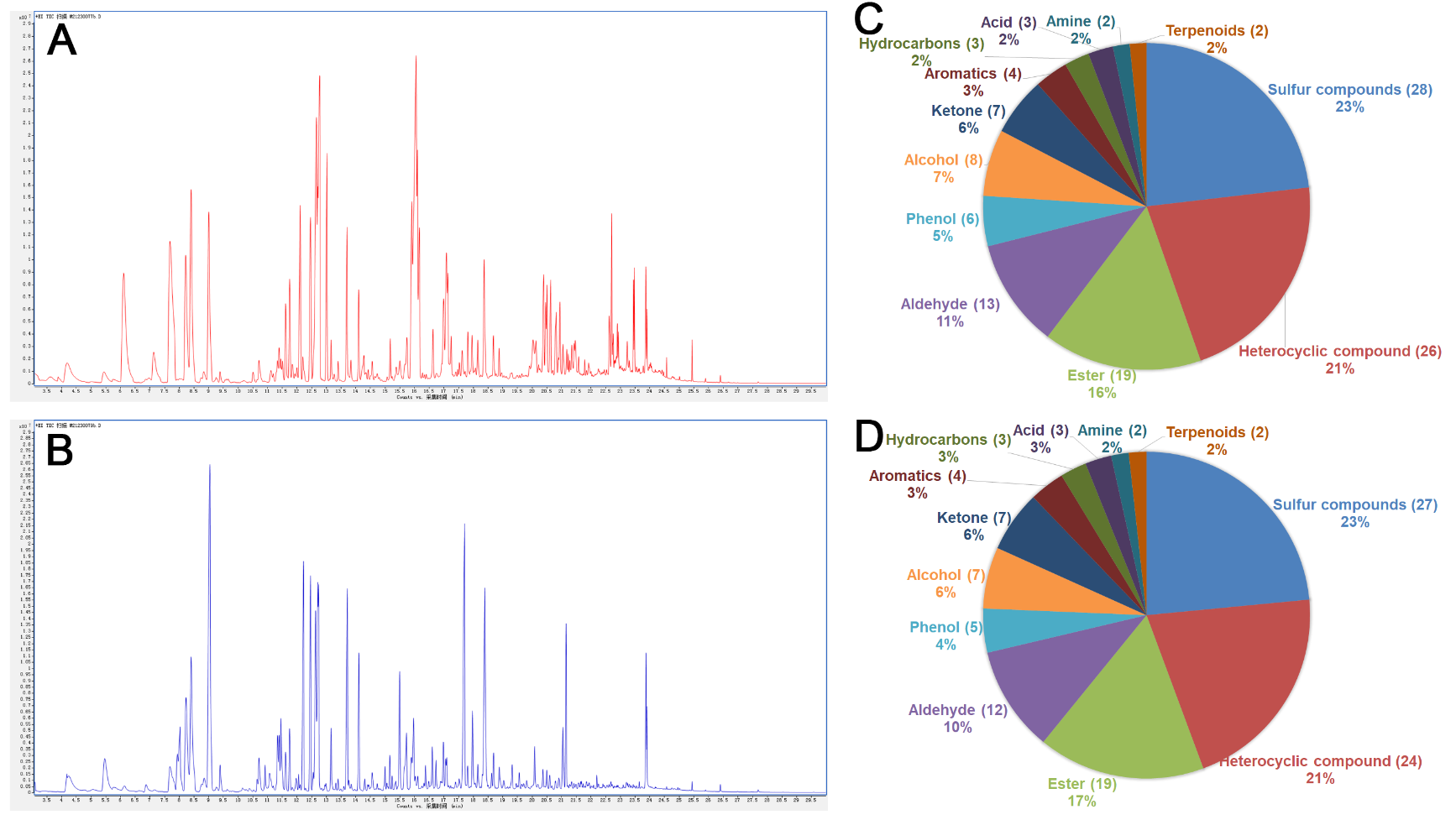


**Figure S2**

**
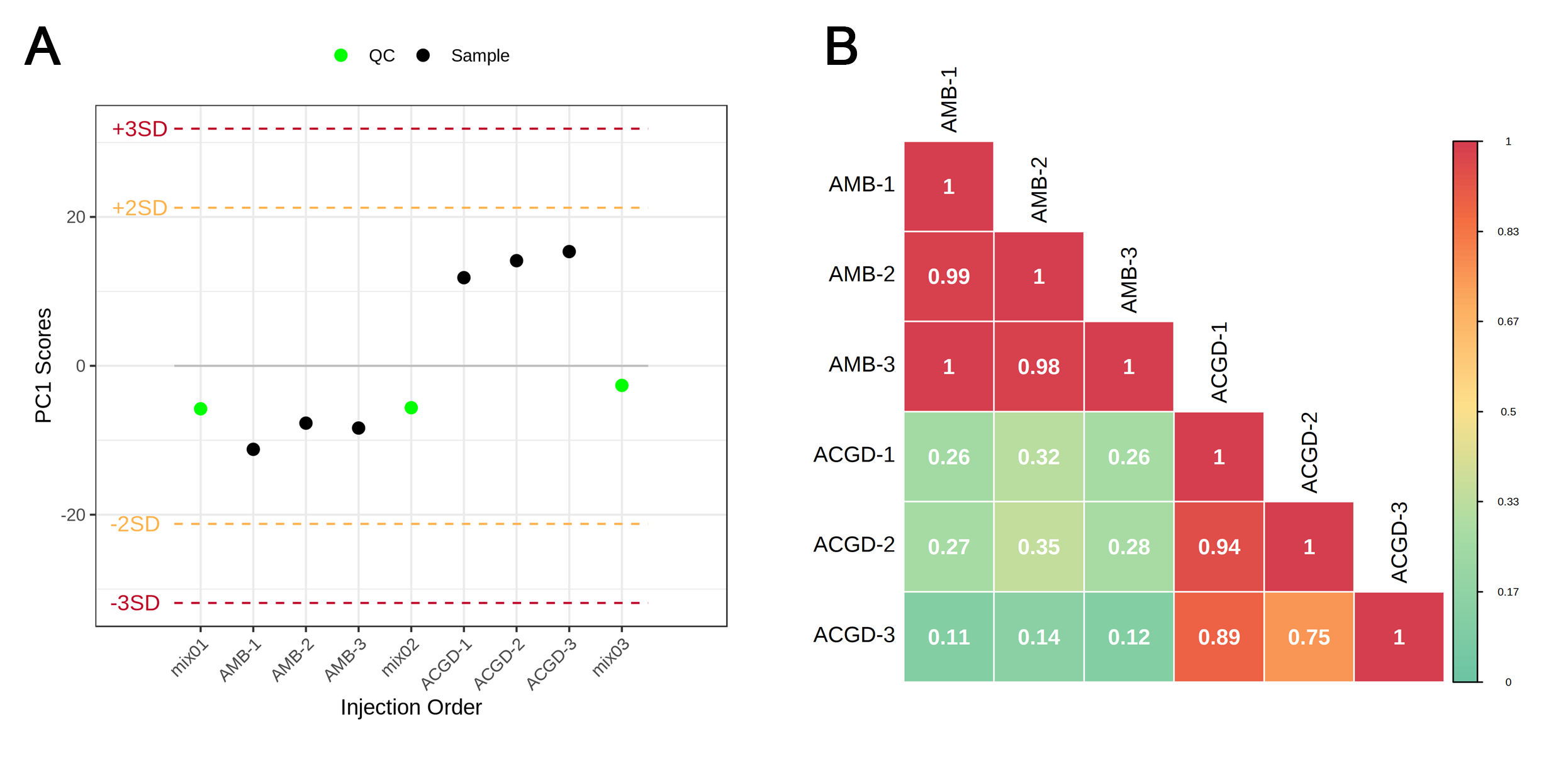
**

**Figure S3**


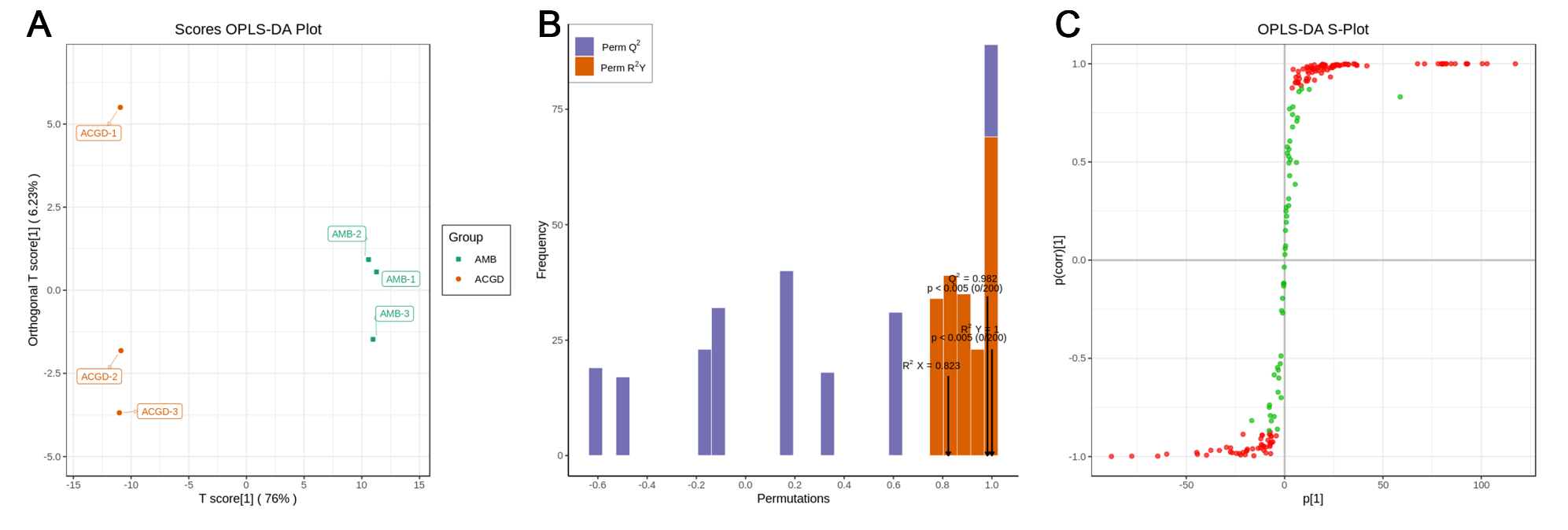


**Figure S4**


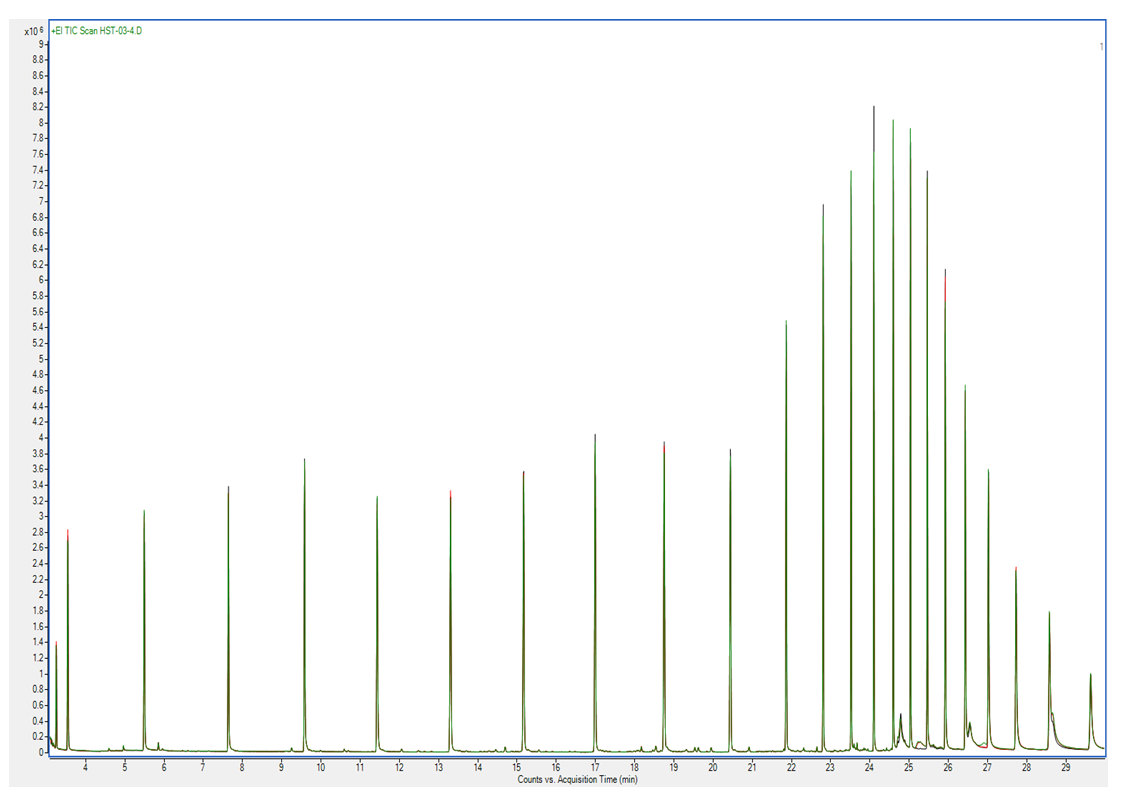


**Figure S5**
